# Targeting OTUD7A-HINT1 deubiquitination activates mTOR signaling for CNS regeneration

**DOI:** 10.1038/s41419-026-09005-4

**Published:** 2026-06-20

**Authors:** Zhen-Gang Liu, Yong-Quan Sun, Lai-Yang Zhou, Ze-Yu Liu, Ling-Wei Zhao, Feng-Quan Zhou, Bo-Yin Zhang, Chang-Mei Liu

**Affiliations:** 1 Department of Orthopaedics, China-Japan Union Hospital of Jilin University, Changchun, 130033 China; 2 Key Laboratory of Organ Regeneration and Reconstruction, Institute of Zoology, Chinese Academy of Sciences, 100101 Beijing, China; 3 University of Chinese Academy of Sciences, 100049 Beijing, China; 4 Beijing Institute for Stem Cell and Regenerative Medicine, 100101 Beijing, China; 5 Center for Translational Neural Regeneration Research, Sir Run Run Shaw Hospital, Zhejiang University School of Medicine, Hangzhou, 310016 Zhejiang China

**Keywords:** Cellular neuroscience, Epigenetics

## Abstract

Axon regeneration in the central nervous system (CNS) remains limited, imposing severe constraints on functional recovery after injury. Here, we reveal that the deubiquitinase OTU deubiquitinase 7 A (OTUD7A) critically regulates CNS regeneration by modulating histidine triad nucleotide-binding protein 1 (HINT1) stability. OTUD7A stabilizes HINT1 protein through specific removal of K63-linked ubiquitin chains at lysine 7. Screening of the small-molecule deubiquitinase inhibitor PR-619 identified HINT1 as a key ubiquitination-regulated target. Notably, genetic knockdown of *Hint1* alone was sufficient to improve RGC survival and promote optic nerve regeneration, thereby activating mTOR signaling, while PR-619 administration enhanced tissue preservation and axon repair after spinal cord injury. A multi-gene therapeutic strategy further enhanced optic nerve regeneration in the optic nerve crush (ONC) model. These findings identify the OTUD7A–HINT1–mTOR axis as a potential therapeutic target in CNS regeneration.

**Figure:**
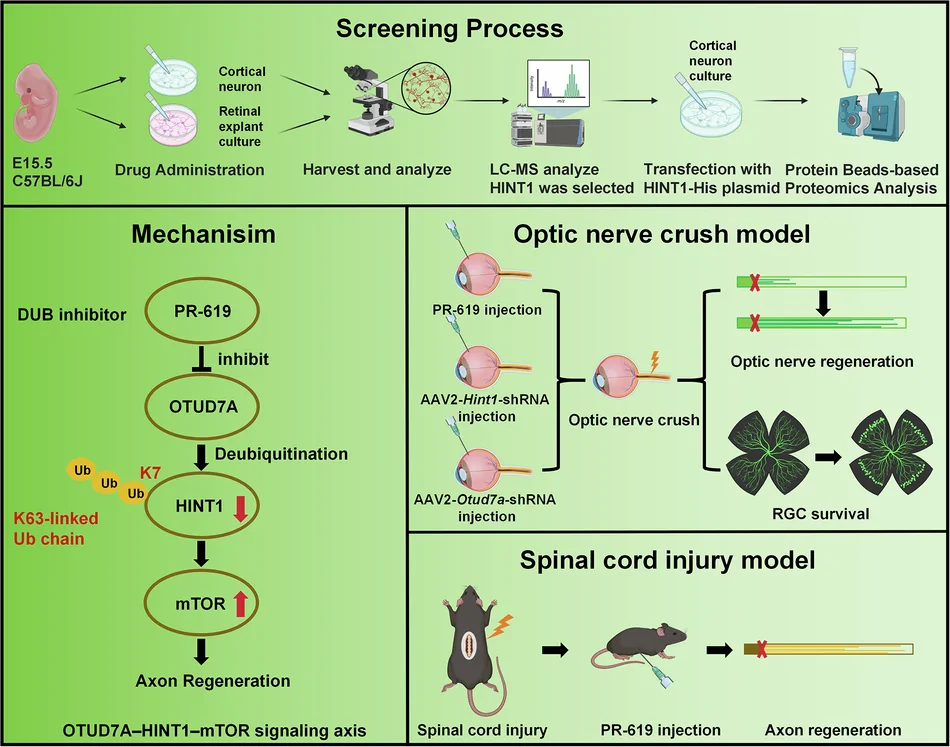

## Introduction

CNS injuries are vulnerable to various traumatic damages, with limited functional recovery that severely impacts the integrity of both function and structure, including stroke [1], optic nerve injury [2, 3], spinal cord injury (SCI) [4, 5], and traumatic brain injury [6]. Axon regeneration failure, compounded by limited treatment options, makes CNS injury repair a major challenge in both research and clinical practice [7, 8]. The mechanisms underlying the restricted axon regeneration in the CNS are partially attributed to the inability of neurons to effectively activate regeneration-associated genes and signaling pathways following injury [9–11]. Recent studies have shown that the deletion of regulatory molecules such as PTEN [12, 13], SOCS3 [10, 14], and REST [10] can substantially enhance regenerative capacity through transcription factor regulation and gene reprogramming. Epigenetic modifications, including EZH2-catalyzed H3K27me3 methylation [15], TET1/2-mediated DNA demethylation, and reactivation of developmental pathways [16], have been shown to promote regeneration. Additionally, the activation of *Opa1*, *Mfn2* [17], and *Optn* [18] improves mitochondrial transport efficiency along microtubules and enhances ATP supply to the injury site, thereby promoting axon regeneration. These studies suggest that gene therapy still holds considerable promise for axon regeneration, but numerous avenues in this field remain unexplored.

Ubiquitination, as an important post-translational modification, plays a vital role in protein quality control by efficiently removing misfolded or damaged proteins, thus maintaining cellular homeostasis [19–21]. Targeting ubiquitination pathways has emerged as a promising therapeutic strategy for various CNS disorders. Ubiquitination involves a cascade of reactions catalyzed by three types of enzymes (E1, E2, E3), making it a key mechanism for regulating numerous cellular activities [22]. Ubiquitin signaling is tightly controlled by deubiquitinases (DUBs), which can counteract ubiquitin attachment. There are only about 100 deubiquitinases in humans, and their small number reflects their powerful function [23, 24]. Disruption of DUB function has been implicated in several diseases, particularly cancer, neurodegenerative disorders, and autoimmune diseases [25–29]. For instance, USP14 regulates ubiquitin cycling at synapses and NMJ development [30, 31]; UCHL1 maintains monoubiquitin for neuronal health [32, 33] and USP33 promotes axon growth by stabilizing Robo1 via deubiquitination [34, 35]. Although the functions of a few deubiquitinating enzymes in the CNS have been identified, the lack of systematic screening means that many potential regulators remain unexplored.

Recent years have seen the emergence of small molecules that modulate the ubiquitination process, such as proteasome inhibitors (e.g., MG132) [36], E3 ligase inhibitors (e.g., MLN4924) [37], and deubiquitinase inhibitors (DUB Inhibitors) [21, 38]. These compounds, especially DUB inhibitors, represent a promising class of drugs that can suppress DUB activity and regulate cellular processes [39]. PR-619, a broad-spectrum DUB inhibitor, has shown potential in enhancing cellular ubiquitin signaling by inhibiting DUB activity and increasing the accumulation of polyubiquitinated proteins. While PR-619 has demonstrated efficacy in cancer therapy [40, 41], its role in CNS injury repair has not been previously explored.

In this study, we identified PR-619 as a broad-spectrum deubiquitinase inhibitor that promotes CNS repair, including ONC and SCI. PR-619 enhanced neurite outgrowth in vitro and, in vivo, protected RGC survival and promoted axon regeneration after ONC. In addition, PR-619 also promoted axon sprouting and partial functional recovery in a SCI model. Proteomics revealed Histidine Triad Nucleotide Binding Protein 1(HINT1) as a key target acting via the mTOR pathway. Further screening identified OTUD7A as an upstream regulator that directly interacts with HINT1 and removes K63-linked ubiquitin chains at lysine 7 (K7). This study provides new theoretical insights and potential therapeutic targets for treating CNS injuries, advancing the development of specific DUB inhibitors for clinical use.

## Materials and methods

### Primary cortical neuron culture

Cortical neurons were prepared from E15.5 C57BL/6 J mice. The brains were rapidly removed and placed in ice-cold phosphate-buffered saline (PBS). The cortical tissue was dissected under a stereomicroscope, and then transferred to a 15 mL conical tube containing ice-cold PBS. After mechanical dissociation, the tissue was digested with 0.25% trypsin (Invitrogen, 25200056) at 37 °C for 10 min. The digestion reaction was stopped by adding an equal volume of culture medium, and the mixture was gently pipetted to further dissociate the cells. After standing for a few minutes, the supernatant was retained, and resuspended in neurobasal medium (Gibco, 21103049) supplemented with 2% B-27 (Gibco, 12587010), 1% GlutaMAX (Gibco, 35050061), and 1% penicillin/streptomycin (Gibco, 15140122). Cortical neurons were plated onto glass-bottom dishes coated with poly-D-lysine (PDL; Sigma-Aldrich, P6407) for neurite outgrowth assays. Neurons were cultured at 37 °C in a humidified atmosphere with 5% CO_2_ to promote neurite growth. The culture medium was changed every 2–3 days. In the neurite outgrowth assay, the total neurite length of each neuron was measured using ImageJ software. Fluorescent images were captured using a fluorescence microscope, and image segmentation was performed using the threshold tool. All neurite branches were traced from the neuronal soma, and the total length of each branch was quantified.

### Retinal explant culture

Postnatal day 0 (P0) C57BL/6 J mice were used for retinal explant culture. Prior to the experiment, retinal explant culture medium (Neurobasal + 2% B27 + 1% GlutaMAX + 1% P/S) was pre-warmed to 37 °C. All procedures were carried out under a biosafety cabinet in compliance with GLP standards. After euthanizing the mice, the eyes were quickly removed and placed in a pre-chilled sterile petri dish. The retina was dissected into 1×1 mm² pieces, which were then transferred to poly-D-lysine-coated glass bottom dishes (MatTek Corp., P35G-1.5-14-C), and the appropriate amount of Neurobasal culture medium was added. The culture medium was replaced the next day, adding small molecules or DMSO (control). On days 5 or 7, the tissues were collected and processed for immunostaining. The dishes were placed in a humidified incubator (5% CO_2_, 37 °C), maintaining an environmental humidity of over 95%. The culture medium was changed every 72 h, taking care to avoid excessive disturbance of the cells.

### Animals

All animal experiments were approved by the Animal Ethics Committee of the Institute of Zoology, Chinese Academy of Sciences, and were conducted following national ethical guidelines for animal care and use (IOZ-IACUC-2022-137). Wild-type (C57BL/6 J) mice (both male and female, aged 5–8 weeks, *n* = 10 per group) were randomly divided into different experimental groups. All mice were housed in a specific-pathogen-free facility with a 12-h light/dark cycle, and had ad libitum access to food and water. Female *Utx*^*f/f*^ (*Kdm6a*^*f/f*^) mice (Kdm6atm1c(EUCOMM)Jae) with loxP sites flanking exon 3 of the Kdm6a gene and *Pten*^*f/f*^ mice (B6.129S4-Ptentm1Hwu/J) with loxP sites flanking exon 5 of the *Pten* gene were purchased from The Jackson Laboratory.*Utx*^*f/f*^*/Pten*^*f/f*^ mice (*n* = 10 per group) were bred in-house by the author, generated through the crossing of *Utx*^*f/f*^ and *Pten*^*f/f*^ transgenic mice, and were used for the experimental protocols described in the methods section.

### Intravitreal injections and optic nerve crush

Under anesthesia, optic nerve injury was performed as previously described [12, 42]. Briefly, the optic nerve was exposed intra-orbitally, and fine forceps were used to crush the optic nerve for 5 s, approximately 1 mm behind the optic disc. Following ONC, mice received intravitreal injections of 1–2 μL of PR-619 or 10% DMSO as a control. In the viral experiments, 1–2 μL of AAV2/2 virus (5 × 10^12^ GC/mL) was injected into the right eye using a glass micropipette. Fourteen days post-injection, ONC was performed. To assess axon regeneration, 1.5 μL of Alexa Fluor 555-conjugated cholera toxin subunit B (CTB-488, CTB-555; 1 μg/μL, Thermo Fisher Scientific) was injected intravitreally for anterograde labeling. After 48 h, mice were transcardially perfused with ice-cold 4% paraformaldehyde (PFA) prior to sacrifice. After perfusion, the right optic nerve and bilateral retinas were harvested and post-fixed in 4% paraformaldehyde (PFA) overnight at 4 °C.

### Retinal processing

After fixation in 4% PFA overnight at 4 °C, the whole retina was radially cut into a petal shape for whole mount retinas immunostaining. For slicing immunostaining, frozen retinal sections (20 μm thick) were obtained using a cryostat. After washing the sections or whole mount retinas with PBS at room temperature for 15 min, the tissues were blocked in PBS containing 0.3% Triton X-100, 2% BSA, and 5% goat serum for 1–2 h. The tissues were then immunostained overnight at 4 °C with the RBPMS antibody. After staining, the sections or retina samples were incubated at room temperature for 2 h with Alexa Fluor-conjugated secondary antibodies, with all antibodies diluted in the blocking solution.

### Optic nerve processing

After perfusion with 4% PFA, the optic nerves were harvested and post-fixed in 4% PFA overnight at 4 °C. The optic nerves were then dehydrated in a graded series of tetrahydrofuran (THF, Sigma-Aldrich), with each concentration (50–100%) for 20 min. Subsequently, the optic nerves were immersed in a 2:1 mixture of benzyl benzoate/benzyl alcohol (BBBA, Sigma-Aldrich) solution until the optic nerves became fully transparent. To analyze axon regeneration, Z-stacked (step size: 2 μm) and tiled fluorescent images were acquired using a Zeiss LSM 880 confocal microscope with a 20× objective. All images were Z-projected to maximal intensity for quantification. To quantify the number of regenerating axons in each optic nerve, every five consecutive sections were maximum projected to generate a series of 10 μm-thick optical slices. At each 250 μm interval from the injury site, the number of regenerating axons was counted and summed across all Z-projected images.

### Plasmid construction

The shRNA sequences of *Otud7a* and *Hint1* were cloned into the pAAV-U6-CAG-GFP vector. Plasmids for *Otud7a* and *Hint1* were obtained from MiaoLingPlasmid (China) for PCR amplification. The *Hint1* gene, with restriction enzyme sites, was subcloned into the pAAV-EGFP (Addgene, 37825) AAV construct under the CAG promoter, resulting in the generation of AAV-CAG-*Hint1*. The knockdown and overexpression efficiencies of the plasmids were confirmed using qPCR. Neuro2a cells (ATCC, CCL-131) were cultured on a 12-well plate to 70–80% confluence. The cells were transfected with 1.5 μg of plasmid DNA using Lipofectamine 3000 for 48 h according to the manufacturer’s instructions. Total RNA was extracted using Trizol reagent (Invitrogen) and then reverse-transcribed into cDNA using the One-Step gDNA Removal and cDNA Synthesis Kit (Tran, AT311-03). RT-PCR was performed in triplicate with Hieff TM qPCR SYBR Green Master Mix (YEASEN), with *Gapdh* serving as the reference gene. The primers used for RT-PCR are listed in Table S1. Plasmids containing *Hint1* mutants such as *Hint1*^K7R^, *Hint1*^K21R^, *Hint1*^K25R^, *Hint1*^K30R^, *Hint1*^K57R^, *Hint1*^K58R^, *Hint1*^K82R^, *Hint1*^K83R^, *Hint1*^K91R^ were constructed by PCR amplification. All constructs were verified by DNA sequencing. HA-UB (WT, K48R, K63R) plasmids (P69179, P69176, P69178) were obtained from MiaoLing Plasmid (China). The control AAV-PLAP plasmid was purchased from Vigene Biosciences (Jinan, China). All AAVs were packaged into serotype 2/2 AAVs (titers: 5 × 10¹² GC/mL) by Delivectory Biosciences (Beijing, China).

### Immunostaining

For retinal sections, mice were anesthetized and transcardially perfused with PBS followed by 4% PFA. Retinal sections (20 μm thick) were incubated in primary antibodies overnight at 4 °C, including anti-RBPMS (1:500, PhosphoSolutions), anti-HINT1 (1:500, Abcam), and anti-pS6 (1:500, Cell Signaling). Secondary antibodies conjugated to Alexa Fluor dyes were used for visualization. For whole mount retinas, retinas were cut radially and incubated with primary antibodies as described. The brain tissue was fixed using 4% PFA overnight at 4 °C. Next, the brain was placed in a 30% sucrose solution for dehydration, and the tissue was left overnight for two nights. After dehydration, the brain tissue was transferred to a cryostat (Leica SM2010R) for sectioning, with a thickness set to 40 μm. After sectioning, the slices were washed with PBS to remove residual sucrose solution. Following mounting, the slices were observed under a microscope for tracing analysis, combined with CTB-555 labeled optic nerves. For spinal cord sections, after deparaffinization or cell fixation, the tissue was washed with PBS and permeabilized with 0.3% Triton X-100. The tissue was then blocked with an immunofluorescence blocking solution, ensuring the entire process was carried out in the dark. The primary antibody was incubated overnight at 4 °C. The next day, the sections were incubated with the corresponding fluorescent secondary antibodies and stained with DAPI to label the nuclei. Immunofluorescence results were observed under a fluorescence microscope.

### LC-MS analysis

Cortical neurons from E15.5 C57BL/6 J mice were first cultured in 10 cm plates for 3 days. After treatment with 1 μM PR-619 or DMSO for 48 h, the culture medium was discarded. Cells were then lysed using RIPA buffer (Beyotime Biotechnology, P0013B) supplemented with protease inhibitor PMSF (Beyotime Biotechnology, ST506) for 30 min. The cell lysates were centrifuged at 13,000 rpm for 15 min at 4 °C. Protein concentrations were quantified using the BCA Protein Assay Kit (Beyotime Biotechnology, P0012). The samples from the PR-619 and DMSO groups were sent for LC-MS/MS analysis. LC-MS/MS analysis was performed using an Orbitrap Astral mass spectrometer coupled with a Vanquish Neo UHPLC system. Peptides were separated using an HPLC column and eluted over 8 min with a linear gradient. Data were acquired using the Orbitrap instrument in DIA mode, with a full scan from 350 to 1500 m/z, followed by MS/MS scans. The data were analyzed using DIA-NN software, and database searching was performed using UniProtKB. Common modifications such as carbamidomethylation of cysteine and acetylation at the N-terminal of proteins were considered. The results were filtered with a false discovery rate (FDR) of less than 1%.

### Bioinformatics analysis

Bioinformatic analysis was performed using Microsoft Excel and R statistical computing software. Hierarchical clustering analysis and volcano plots were generated using the R statistical language. Sequence annotation was carried out by extracting information from UniProtKB/Swiss-Prot, Kyoto Encyclopedia of Genes and Genomes (KEGG), and Gene Ontology (GO). GO and KEGG enrichment analyses were performed using Fisher’s exact test, and false discovery rate (FDR) correction for multiple testing was applied. GO terms were categorized into three groups: biological process (BP), molecular function (MF), and cellular component (CC). Enriched GO and KEGG pathways were considered nominally statistically significant if the Fisher’s exact test p-value was <0.01. Protein-protein interaction (PPI) networks were constructed using the STRING database and visualized with Cytoscape software.

### Western blot

Western blotting was performed according to the standard protocol. Protein samples were first extracted, and protein concentrations were determined using a BCA protein assay kit. The proteins were then separated by SDS-PAGE and transferred onto PVDF membranes. After transfer, the membranes were blocked with 5% skim milk at room temperature for 1 h, followed by overnight incubation at 4 °C with primary antibodies diluted to the appropriate concentration. The following day, the membranes were washed three times with TBST buffer for 15 min each and then incubated with HRP-conjugated secondary antibodies for 2 h. After incubation, the membranes were developed using an enhanced chemiluminescence detection system to visualize and record the protein bands.

### CO-IP/IP-MS

To perform immunoprecipitation, HEK293T cells were first seeded on a 10 cm culture dish and cultured to 70–80% confluence. The cells were then transfected with 5 μg of various plasmid constructs using PEI. After 48 h of transfection, the cells were treated with 10 μM MG132 (Selleck, S2619) for 6 h. Subsequently, the cells were lysed with NP40 lysis buffer (85 mM KCl, 5 mM PIPES, 0.5% NP40) containing PMSF protease inhibitor for 30 min. After lysis, the cell lysate was centrifuged at 13,000 rpm for 15 min at 4 °C, and the supernatant (whole-cell lysate) was collected. The whole-cell lysate was then incubated with Protein A/G-agarose beads (Santa Cruz, SC-2003) at 4 °C for 1 h for pre-treatment, and a portion of the lysate was retained as the input sample. The supernatant was then incubated overnight at 4 °C with the relevant primary antibodies, IgG as negative control. The next day, Protein A/G-agarose beads were added for an additional 6 h of incubation. The precipitates were washed three times with immunoprecipitation buffer. For the co-IP experiment, the sample was denatured and boiled for 5 min. For IP-MS, beads were directly used for MS detection.

### Spinal cord injury model

SCI was induced by performing a laminectomy at the T8-T10 vertebral levels. An impactor was then used to deliver a controlled injury to the spinal cord. The impact was applied at a depth of 2 mm and at a velocity of 1 m/s, following the manufacturer’s guidelines. Successful SCI was confirmed by the visible injury site and the presence of spontaneous limb movements and tail twitching. After the injury, the wound was carefully sutured in layers, and the animals were returned to their cages for recovery. To aid in urinary function recovery, manual bladder expression was performed three times daily until the mice resumed spontaneous urination. Mice in the sham group underwent the laminectomy procedure only, with all other steps mirroring those of the SCI group.

### Statistical analysis

All in vitro and in vivo data in this study are presented as means ± standard error of the mean (SEM). The sample sizes between groups were determined with reference to previous literatures in the same research field for adequate power to detect a pre-specified effect. For Western blot analysis, each experiment was independently repeated at least three times three biological replicates and two technical replicates per sample with consistent results. For comparisons between two groups, an unpaired two-tailed Student’s *t* test was used. For comparisons involving three or more groups, one-way analysis of variance (ANOVA) was performed, followed by Tukey’s multiple comparisons test to evaluate statistical significance. A *P* value of less than 0.05 was considered statistically significant. Statistical analysis was conducted using GraphPad Prism (GraphPad Software, La Jolla, CA). No statistical method was used for estimating sample size for animal experiments.

## Results

### PR-619 promotes RGC survival and axon regeneration

To assess the effect of global ubiquitination on axon regeneration, we screened proteasome and deubiquitinase inhibitors in primary mouse cortical neurons. PR-619, a deubiquitinase inhibitor, markedly promoted neurite outgrowth (Figs. S1A, B, S2A), whereas MG132 did not (Fig. S2B, C). At 0.5 μmol/L optimal concentration, PR-619 significantly increased neurite length (Fig. S1B, D). Using a retinal explant model (Fig. S1C), PR-619 also enhanced neurite regeneration at days 5 and 7 (Fig. S1E, F). Thus, PR-619 promotes neurite outgrowth in both cortical neurons and RGCs.

To evaluate the potential of PR-619 in optic nerve injury repair, we first conducted a toxicity screening to determine safe concentration levels for subsequent experiments (Fig. S3A). In this toxicity screening, we found that PR-619 concentrations between 5 and 50 μM did not significantly affect RGC survival, whereas concentrations of 100–200 μM led to a notable reduction in RGC survival (Fig. S3B, C). Additionally, we assessed the inflammatory response in the retina following PR-619 treatment. Immunofluorescence staining showed that the expression levels of microglial markers (CD68 and IBA1) remained unchanged in the PR-619-treated group (Fig. S4). To verify the pharmacological effects and inhibitory efficiency of PR-619 in the experimental system of this study, we performed immunofluorescence co-staining for total ubiquitin and RBPMS in RGCs, the results showed that the ubiquitin fluorescence intensity in RGCs was significantly increased after PR-619 treatment (Fig. S3D, E), directly demonstrating that PR-619 effectively inhibits DUB activity in RGCs with definite inhibitory efficiency. Notably, PR-619 had no significant effect on the ubiquitination level in the inner nuclear layer (INL) of the retina (Fig. S3F, G), which further reflects the cell-type-specific effect of PR-619 on RGCs and reduces the possibility of non-specific off-target toxicity in other retinal cells.

**Fig. 1:**
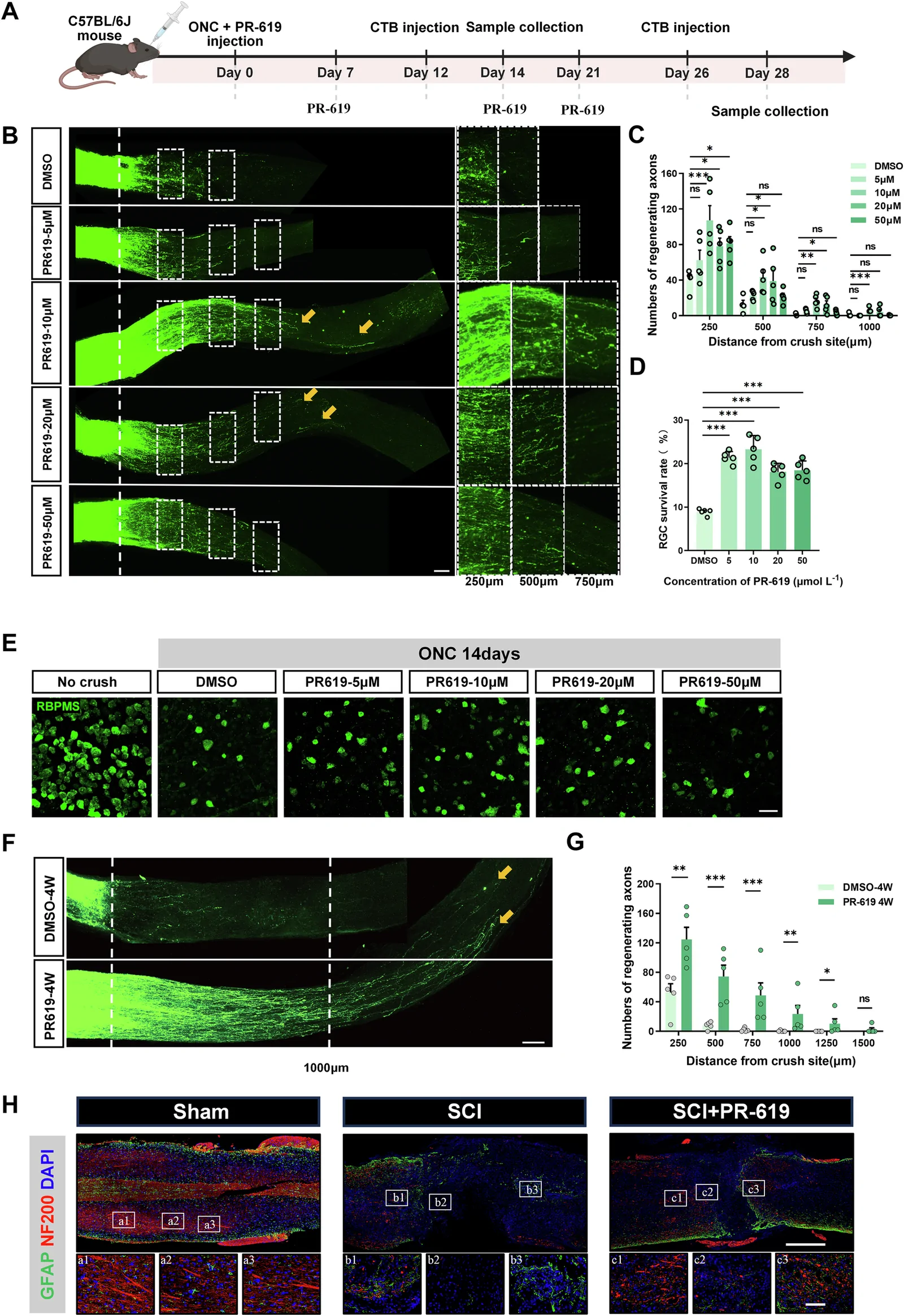
PR-619 promotes axon regeneration after central nervous system injury. **A** Experimental timeline of ONC with PR-619 treatment.6-week-old mice received ONC and PR-619 injection on Day 0, followed by PR-619 injections on Days 7, 14, and 21, CTB-555 injections on Days 12 and 26, and tissue harvest on Days 14 and 28. **B** Representative images of CTB-555 labeled regenerating axons in the optic nerve at 14 days post-ONC, showing enhanced regeneration with graded PR-619 concentrations (5–50 μM) compared with DMSO. Dashed line indicates crush site; insets show axons at 250, 500, and 750μm from the lesion. Scale bar, 100 μm. **C** Quantification of regenerating axons at increasing distances (250–1000 μm) to the lesion in different PR-619 treatment groups (*n* = 5 animals, data are means ± SEM. One-way ANOVA followed by Tukey’s multiple comparisons test, ns no significant, *p < 0.05, **p < 0.01, ***p < 0.001). **D** Quantification of RGC survival at 14 days post-ONC in DMSO- and PR-619-treated animals, showing a significant increase in survival at PR-619 concentrations of 5–50 μM compared to the DMSO group (*n* = 5 animals, data are means ± SEM. One-way ANOVA followed by Tukey’s multiple comparisons test, ***p < 0.001). **E** Representative RBPMS immunostaining of RGCs (green) in uninjured, DMSO-treated, and PR-619-treated eyes (5–50 μM) at 14 days post-ONC. Scale bar: 100 μm. **F** Representative images of CTB-555 labeled regenerating axons at 4 weeks post-ONC. The dashed line indicates the crush site. Scale bar: 50 μm. **G** Quantification of regenerating axons at increasing distances (250–1500 μm) from the lesion at 4 weeks post-ONC, showing significantly higher regeneration in PR-619-treated animals (*n* = 5 animals, data are means ± SEM. Unpaired two-tailed *t* test, ns no significant, **p < 0.01, ***p < 0.001). **H** Immunofluorescence: GFAP (green), NF200 (red), and DAPI (blue) staining of spinal cord sections. Compared with the SCI group, PR-619 markedly increased NF200-positive axonal density and continuity, while reducing GFAP reactivity. Upper panels show overviews, Scale bar: 1000 μm; boxed regions correspond to enlarged images a1–a3, b1–b3, and c1–c3. Scale bar: 100 μm.

In the ONC model, 6-week-old mice underwent ONC followed by PR-619 treatment, with histological analysis conducted at various time points (Fig. 1A). The results showed that PR-619 (5–50 μM) significantly increased both axon regeneration (250–1000 μm distal to crush) and RGC survival (Fig. 1B–E). A 4-week experiment confirmed long-distance regeneration with 10 μM PR-619 (Fig. 1F, G). However, while PR-619 enhanced RGC survival during the first two weeks, this effect diminished by week 4 (Fig. S5A, B). These results suggest that while PR-619 supports long-term axon regeneration in the ONC model, its protective effect on RGC survival is limited over time. In contrast, MG132 showed no regenerative or survival benefits (Fig. S2D–G). Additionally, we evaluated the effects of PR-619 in an NMDA-induced retinal injury model. Results revealed a marked increase in RGC survival at 14 days post-NMDA injury in the PR-619-treated group (Fig. S5C–E).

### PR-619 enhances axon regeneration after SCI

To assess the therapeutic effects of PR-619 on spinal cord injury, we conducted histological staining, immunofluorescence analysis, and behavioral assessments (Fig. S6A). HE staining revealed significant tissue cavitation and disorganized architecture in the SCI group, while PR-619 treatment notably reduced lesion size, preserved structural continuity, and maintained more intact parenchymal tissue. Nissl staining showed a dramatic decline in surviving neurons and the dissolution of Nissl bodies in the SCI group. In contrast, PR-619 treatment led to a significant increase in neuronal survival per unit area, highlighting its strong neuroprotective effects. Similarly, LFB staining revealed extensive demyelination after SCI, while PR-619 preserved myelin structures and alleviated the extent of demyelination (Fig. S6B). Immunofluorescence analysis further corroborated these observations. Treatment with PR-619 increased the density and continuity of NF200 positive axons, accompanied by a reduction in GFAP positive astrocytic scarring, suggesting that PR-619 promotes axon regeneration and attenuates glial scar formation (Fig. 1H). Additionally, Masson’s trichrome staining of hindlimb skeletal muscle revealed significant muscle fiber atrophy and fibrosis following SCI. These pathological alterations were notably ameliorated by PR-619, demonstrating its ability to protect neuromuscular function (Fig. S6D). PR-619 treatment significantly improved motor function, with higher BMS scores and greater maximum supporting angles compared to SCI controls (Fig. S6E, F). Remarkably, improvements in function were observed as early as day 7 and persisted throughout the observation period, demonstrating that PR-619 facilitates long-term locomotor recovery after SCI.

### Identification of HINT1 as a downstream target of PR-619 and its role in axon regeneration

**Fig. 2:**
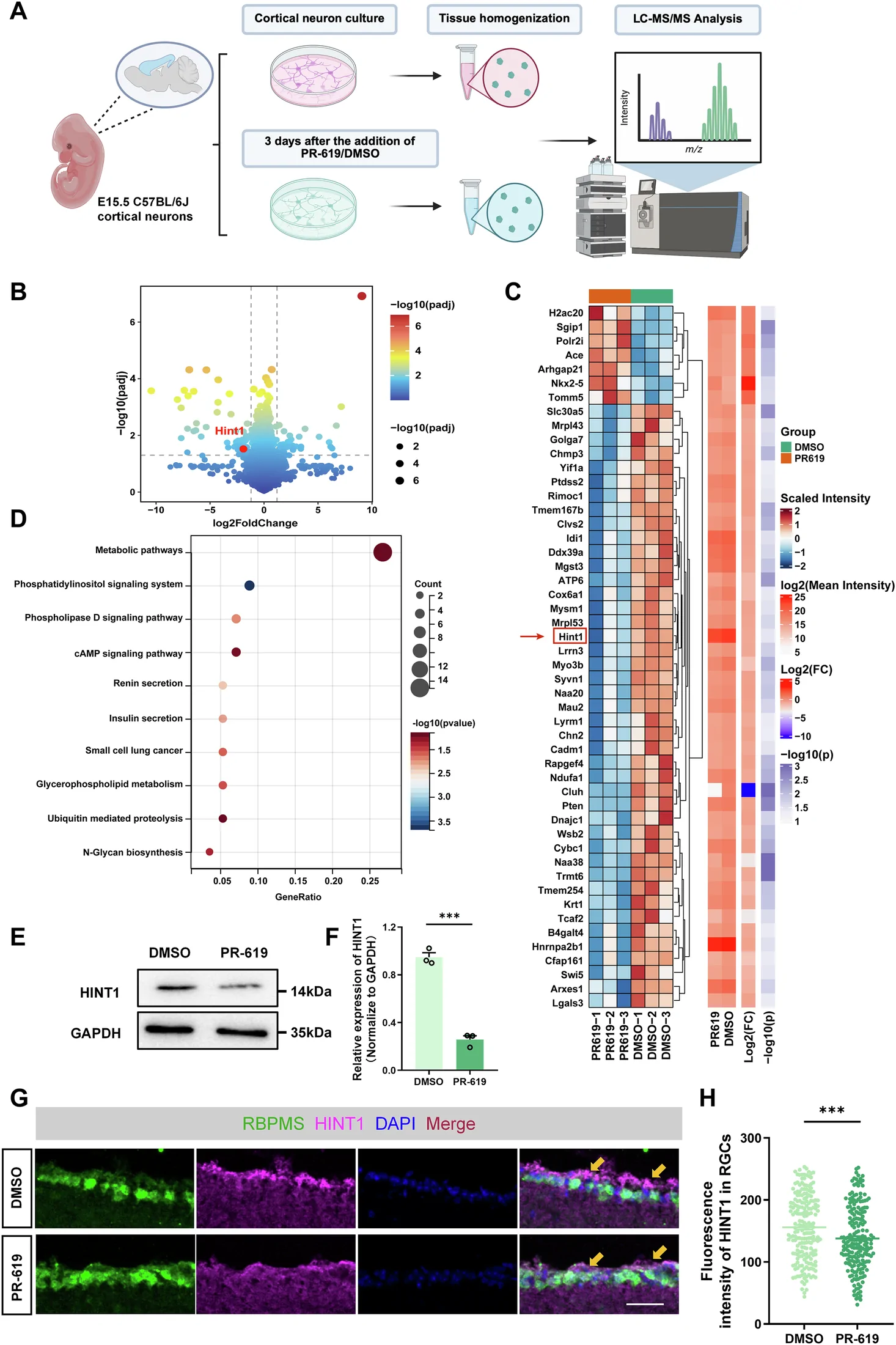
Proteomic analysis identifies HINT1 as a downstream target of PR-619 in cortical neurons. **A** Schematic of the experimental workflow. Cortical neurons were cultured from E15.5 C57BL/6 J mice cortices. After 3 days in culture, cells were treated with PR-619 or DMSO for 48 h, followed by tissue lysis, protein extraction, and LC-MS/MS analysis to identify differentially expressed proteins. **B** Volcano plot displaying differentially expressed proteins in PR-619-treated neurons compared to DMSO controls. Proteins are plotted based on log2 fold change (x-axis) and -log10(*p* value) (y-axis). HINT1 is identified as a significantly downregulated protein, highlighted in red. **C** Heatmap of the top differentially expressed proteins from LC-MS/MS analysis, comparing PR-619 and DMSO treatment. The proteins are clustered based on their expression profiles, with color intensity indicating the scaled intensity of each protein across the samples. HINT1 is highlighted, showing its decreased expression in PR-619-treated neurons. **D** Gene ontology (GO) analysis of the top differentially expressed proteins, showing significant enrichment in metabolic and signaling pathways. **E** Representative western blot showing downregulated HINT1 protein levels in cortical neurons 48 h after treatment with 0.5 µM PR-619, with significantly reduced expression compared to DMSO controls. **F** Quantification of protein level of HINT1 in (**E**) (*n* = 3 independent experiments, data are means ± SEM. Unpaired two-tailed *t* test, *p < 0.05). **G** Representative immunofluorescence images of RGCs labeled with RBPMS (green) and HINT1 (magenta) in DMSO- and PR-619-treated retinas. DAPI (blue) marks the cell nuclei. Scale bar: 50 μm. **H** Quantification of fluorescence intensity of HINT1 in RGCs shown in (**G**) (*n* = 3 animals, n numbers on graph show the number of analyzed cells, data are means ± SEM, unpaired two-tailed *t* test, ***p < 0.001).

To explore how PR-619 promotes axon regeneration, we performed proteomic analysis (Fig. 2A). Differentially expressed proteins were visualized in a volcano plot (Fig. 2B). Notably, based on fold change, p-values, and confidence metrics, HINT1 was identified as being significantly downregulated in the PR-619-treated group (Fig. 2B with HINT1 highlighted in red). Further examination using a heatmap representation (Fig. 2C) revealed that HINT1 was among the most notably altered proteins. GO analysis indicated that the differentially expressed proteins were notably enriched in metabolic and signaling pathways, with a particular focus on the ubiquitin-mediated proteolysis pathway, a process closely associated with ubiquitination (Fig. 2D). This suggests that PR-619 may facilitate neuronal repair and axon regeneration by modulating this critical pathway.

Subsequent western blot analysis confirmed that PR-619 treatment significantly reduced HINT1 protein levels in cortical neurons (Fig. 2E, F). To confirm the relevance of these findings in RGCs, we performed immunofluorescence staining to examine HINT1 expression in RGCs. The immunofluorescence signals for HINT1 exhibited a granular or diffuse distribution in the cytoplasm, with particular enrichment around the cytoplasmic matrix and mitochondria [43, 44]. Additionally, HINT1 was found in the cell nucleus, potentially interacting with the nucleosome or nuclear matrix, and playing a role in transcriptional regulation and other cellular processes (Fig. 2G) [45, 46]. In the retina treated with PR-619, HINT1 expression in RGCs was significantly reduced compared to the DMSO control group (Fig. 2G, H). Collectively, these results position HINT1 as a crucial downstream target of PR-619 in RGCs.

### *Hint1* knockdown promotes axon regeneration and RGC survival after ONC

**Fig. 3:**
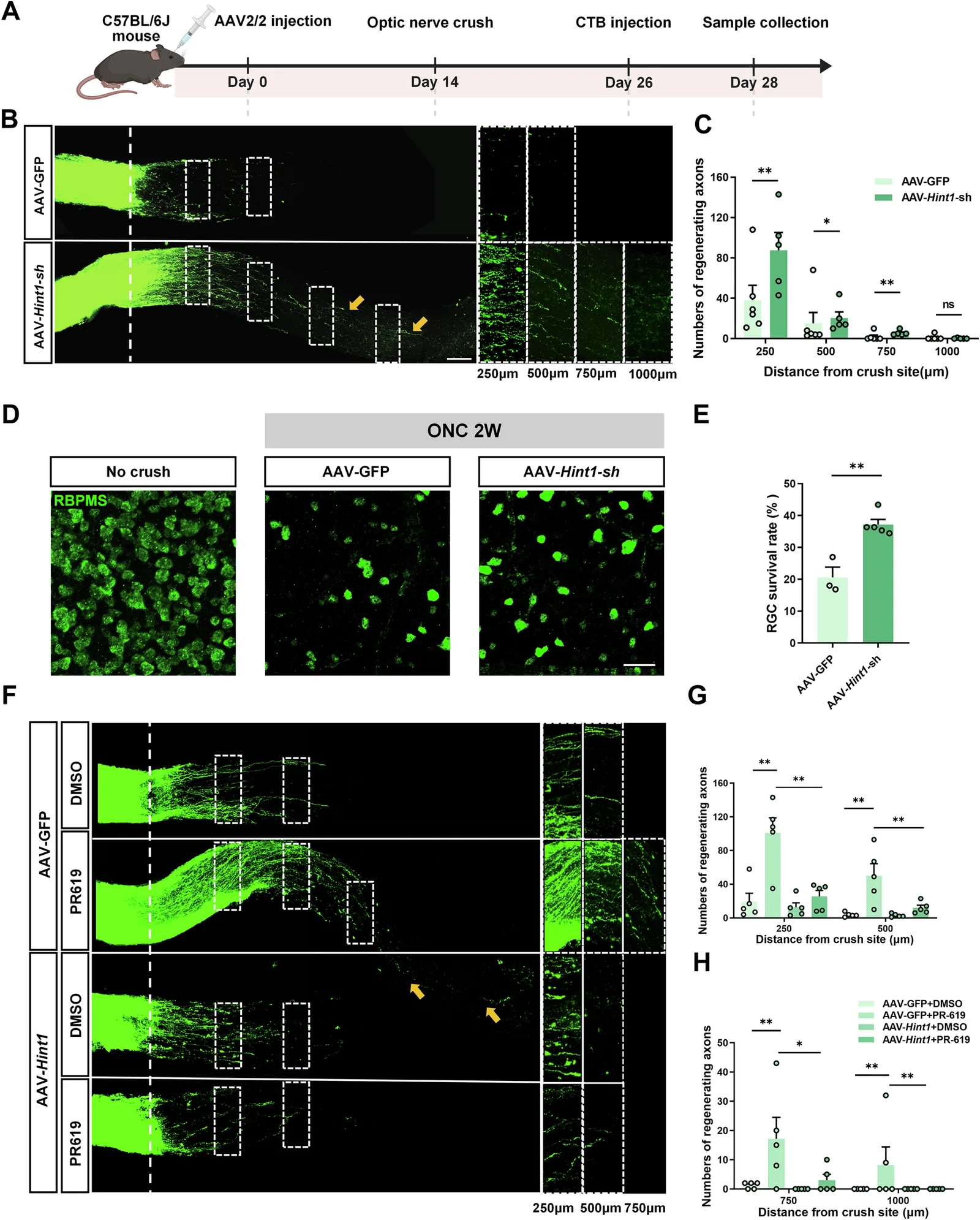
*Hint1* knockdown promotes RGC survival and axon regeneration after ONC. **A** Experimental timeline. Mice received intravitreal injection of AAV-GFP or AAV-*Hint1*-shRNA on Day 0, followed by ONC on Day 14, CTB-555 injection on Day 26, and tissue collection on Day 28. **B** Representative images of CTB-555 labeled axons at 14 days post-ONC. Compared to AAV-GFP controls, AAV-*Hint1*-shRNA treatment significantly increased axon regeneration. Dashed line indicates the crush site; insets show regenerating axons at various distances distal to the lesion. Scale bar: 100 μm. **C** Quantification of regenerating axons, showing that AAV-*Hint1*-shRNA significantly increased axon numbers within 250–750 μm from the lesion site compared to AAV-GFP (*n* = 5 animals per group, data are means ± SEM, one-way ANOVA with Tukey’s post hoc test, ns not significant, *p < 0.05, **p < 0.01). **D** Representative images of RBPMS-labeled (green) RGCs at 14 days post-ONC, showing increased RGC survival in the AAV-*Hint1*-shRNA group compared to the AAV-GFP. Scale bar: 100 μm. **E** Quantification of RGC survival, showing that AAV-*Hint1*-shRNA significantly increased survival compared to AAV-GFP (*n* = 5 animals, data are means ± SEM, unpaired two-tailed *t* test, ***p < 0.001). **F** Rescue experimental design. Mice were intravitreally injected with AAV2-GFP or AAV2-*Hint1*, subjected to ONC on Day 14, and treated with PR-619 or DMSO. CTB-555 was injected on Day 26, and tissues were harvested on Day 28. Scale bar: 100 μm. **G**, **H** Quantification of regenerating axons at increasing distances (250–1000 μm) from the crush site in the AAV-GFP and AAV-*Hint1* groups treated with PR-619 or DMSO, showing that the promotion effect of PR-619 on axon regeneration was completely abolished in the AAV-*Hint1* group (*n* = 5 animals, data are means ± SEM, one-way ANOVA followed by Tukey’s multiple comparisons test, ns not significant, *p < 0.05, ***p < 0.001).

Given that PR-619 is a broad-spectrum deubiquitinase inhibitor, we hypothesized that knocking down *Hint1* in RGCs might further enhance optic nerve regeneration. To test this, we used AAV-*Hint1*-shRNA to knock down *Hint1* in RGCs. The efficiency of AAV transduction in RGCs was confirmed, immunofluorescence images of retinal sections and whole mount retinas revealed approximately 70% co-localization of AAV2 signal with RBPMS (Fig. S8A–C). Additionally, mRNA expression levels of *Hint1* were assessed in N2a cells by qPCR, confirming efficient knockdown and overexpression of *Hint1* (Fig. S8D, E, G, H). CTB-555 labeled optic nerve axons showed that *Hint1* knockdown significantly promoted axon regeneration compared to the AAV-GFP group (Fig. 3A–C). In addition, staining on whole mount retinas indicated that the RGC survival rate in the AAV-*Hint1*-shRNA group was significantly higher than in the AAV-GFP control group (Fig. 3D, E).

To further investigate the role of *Hint1* in PR-619-mediated neuroprotection, we used AAV-GFP or AAV-*Hint1* overexpression viruses to conduct a rescue experiment (Fig. 3F). In the AAV-GFP injected group, the optic nerve regeneration effect in the PR-619 treated subgroup was significantly superior to that in the DMSO-treated subgroup. In contrast, within the AAV-*Hint1*-injected group, the promotion of PR-619 on optic nerve regeneration was markedly attenuated, indicating that *Hint1* expression inhibits PR-619 induced axonal regeneration (Fig. 3G, H). Additionally, we conducted a similar rescue experiment in primary cortical neurons (Fig. S9A). The results demonstrated that *Hint1* overexpression significantly attenuated PR-619 induced axon regeneration (Fig. S9B), which was consistent with the results of in vivo experiments. These findings further support the negative regulatory role of *Hint1* in neuronal regeneration, and collectively confirm that the axon regeneration promoting function of PR-619 is mainly mediated by *Hint1*.

### OTUD7A enhances axon regeneration and RGC survival by regulating HINT1 stability through K63-linked deubiquitination

**Fig. 4:**
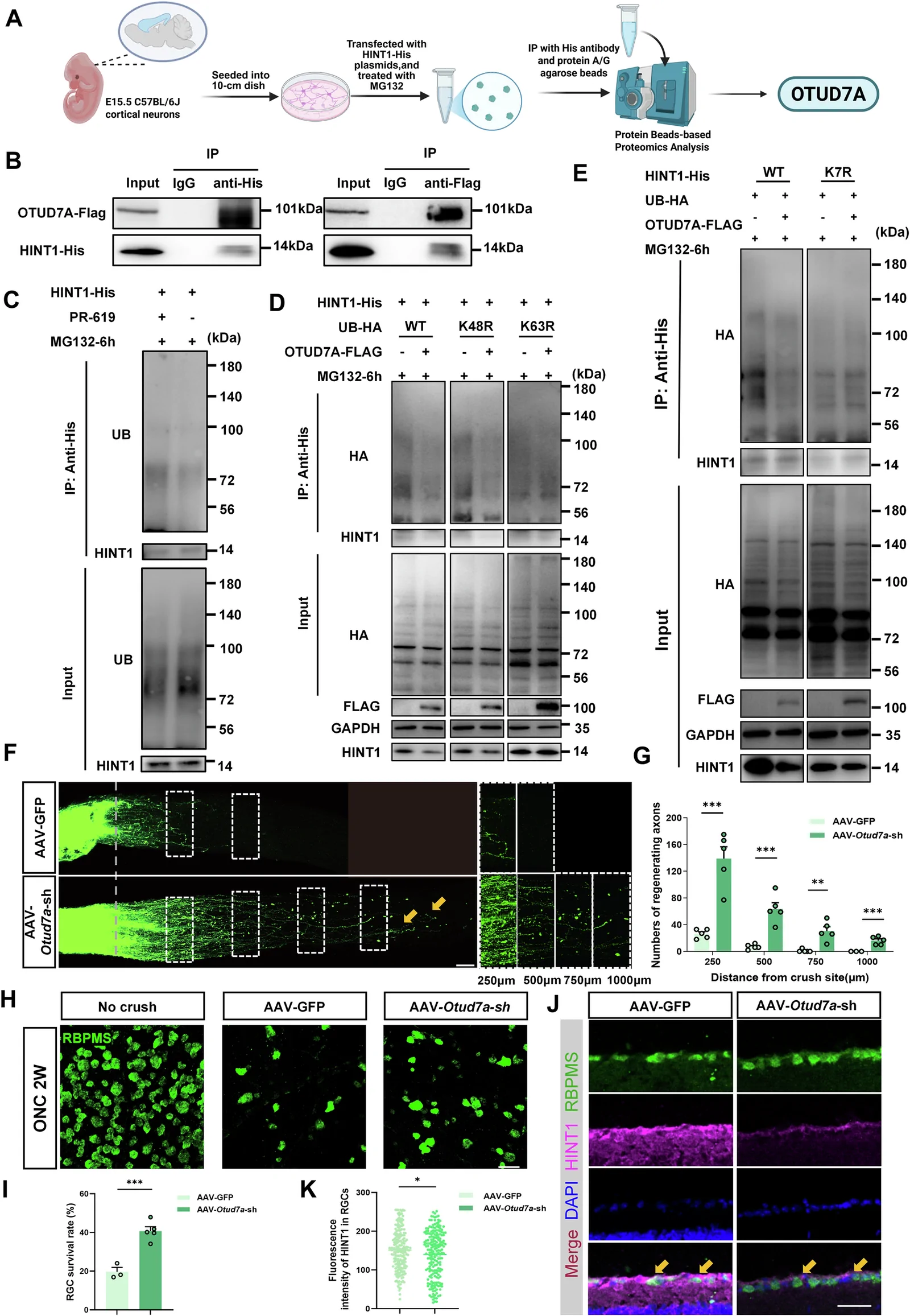
OTUD7A enhances axon regeneration and RGC survival by regulating HINT1 stability through K63-linked deubiquitination. **A** Schematic diagram of the experimental design. Cortical neurons from E15.5 C57BL/6 J mice were transfected with *Hint1*-His plasmids and treated with MG132 for 6 h prior to protein collection. Protein samples were then subjected to immunoprecipitation (IP) using anti-His antibody or protein A/G agarose beads for OTUD7A-related proteomics analysis. **B** Immunoblot analysis of the interaction between *Otud7a*-Flag and *Hint1*-His. Lysates from 293 T cells transfected with *Otud7a*-Flag or *Hint1*-His plasmids were immunoprecipitated with anti-His or anti-Flag antibodies, followed by Western blot detection of OTUD7A and HINT1. Input and IgG controls are included. **C** Effect of PR-619 treatment on *Hint1*-His ubiquitination. Lysates from cortical neurons transfected with *Hint1*-His were treated with PR-619 or control for 6 h, followed by immunoprecipitation with anti-His antibody and Western blot detection of ubiquitin (UB) and HINT1 to assess changes in HINT1 ubiquitination levels. Results show an increase in ubiquitination levels in the PR-619-treated group. **D** Immunoblot analysis of OTUD7A ubiquitin chain linkage preferences. 293 T cells were co-transfected with *Otud7a*-Flag and *Hint1*-His plasmids along with WT Ub-HA or various Ub-HA mutants (K48R, K63R). Immunoprecipitation was performed with anti-His antibody, followed by Western blotting to detect HINT1, HA, and FLAG. Cells were treated with 10 μM MG132 for 6 h prior to collection and ubiquitination assay. **E** Immunoblot analysis of *Otud7a*-Flag effects on *Hint1*-His mutant ubiquitination. 293 T cells were transfected with *Otud7a*-Flag and WT Ub-HA plasmids, along with either WT *Hint1*-His or various *Hint1*-His mutants (K7R) Cells were treated with 10 μM MG132 for 6 h before collection and ubiquitination assay. Results indicate that K7 is the functional site targeted by OTUD7A for ubiquitination. **F** Representative images of CTB-555 labeled axons at 14 days post-ONC. Compared to AAV-GFP, AAV-*Otud7a*-shRNA treatment significantly enhanced axon regeneration. Dashed lines indicate the crush sites; insets show regenerating axons at various distances distal to the lesion. Scale bar: 100 μm. **G** Quantification of regenerating axons at increasing distances from the crush site. The number of regenerating axons was significantly higher in the *Otud7a* knockdown group compared to controls within 250–1000 μm from the lesion site (*n* = 5 animals, data are means ± SEM, one-way ANOVA followed by Tukey’s post hoc test, ns not significant, *p < 0.05, ***p < 0.001). **H** Representative images of RBPMS-stained (green) RGCs showing that *Otud7a* knockdown significantly increased RGC survival 14 days after ONC. Scale bar: 50 μm. **I** Quantification of RGC survival rates. *Otud7a* knockdown significantly improved RGC survival compared to controls (*n* = 5 animals, data are means ± SEM, unpaired two-tailed *t* test, ***p < 0.001). **J** Representative retinal sections showing RBPMS (green), HINT1 (magenta), and DAPI (blue). *Otud7a* knockdown significantly reduced HINT1 expression in RGCs. Scale bar: 50 μm. **K** Quantification of fluorescence intensity, confirming that HINT1 levels were markedly lower in AAV-*Otud7a*-shRNA treated retinas compared to AAV-GFP (*n* = 3 animals, n values indicate the number of analyzed cells, data are means ± SEM, unpaired two-tailed *t* test, ***p < 0.001).

Although PR-619 promotes optic nerve regeneration and RGC survival via *Hint1* downregulation, the specific deubiquitinase that regulates HINT1 stability remains unknown. To identify it, we infected mouse cortical neurons with AAV-*Hint1*-His, treated with MG132, and performed anti-His immunoprecipitation (IP) for proteomic analysis (Fig. 4A). OTUD7A was identified as a direct interactor. Co-IP in 293 T cells co-expressing *Otud7a*-Flag and *Hint1*-His confirmed their interaction (Fig. 4B). To assess the effect of PR-619-induced deubiquitinase inhibition on HINT1, 293 T cells transfected with *Hint1*-His were treated with PR-619 for 24 h, followed by IP and Western blot analysis to detect ubiquitination levels. The results showed that PR-619 treatment increased HINT1 ubiquitination (Fig. 4C). We then analyzed OTUD7A’s ubiquitin chain preferences by co-transfecting 293 T cells with *Otud7a*-Flag, *Hint1*-His, and various ubiquitin mutants. Western blotting revealed that OTUD7A preferentially hydrolyzes K63-linked ubiquitin chains of HINT1 (Fig. 4D). To identify key lysine residues in HINT1 involved in ubiquitination, we used various *Hint1*-His mutants. The results demonstrated that mutation of lysine 7 (K7R) substantially reduced HINT1 ubiquitination by OTUD7A, suggesting that K7 is a critical site for this modification (Fig. 4 E, S10A). Collectively, we identify OTUD7A as a deubiquitinase for HINT1 through direct interaction, catalyzing K63-linked deubiquitination at K7 of HINT1.

To confirm OTUD7A’s role in optic nerve repair, we knocked it down in vivo. Efficient *Otud7a* knockdown was verified by qPCR in N2a cells (Fig. S8F, G, H). OTUD7A knockdown significantly enhanced axon regeneration (Fig. 4F, G) and increased RGC survival (Fig. 4H, I). Immunofluorescence showed that *Otud7a* knockdown reduced HINT1 expression in RGCs (Fig. 4J, K), consistent with its deubiquitinase function. These results highlight OTUD7A as a key regulator of HINT1, and its knockdown not only promotes axon regeneration and RGC survival but also supports the notion that OTUD7A modulates the ubiquitination status of HINT1 to enhance axon regeneration following optic nerve injury.

### *Hint1* knockdown activates the mTOR pathway to promote neuroprotection and axon regeneration after ONC

To investigate whether PR-619 promotes RGC axon regeneration through the activation of classical signaling pathways, we first performed immunostaining to assess the mTOR, GSK3β, and JAK-STAT3 pathways. Our results indicate that PR-619 substantially activated the mTOR pathway, as evidenced by a marked increase in p-S6 positive RGCs compared to the DMSO control group (Fig. S7A, D). However, PR-619 did not significantly alter the activity of the GSK3β or JAK-STAT3 pathways (Fig. S7B, C, E, F).

**Fig. 5:**
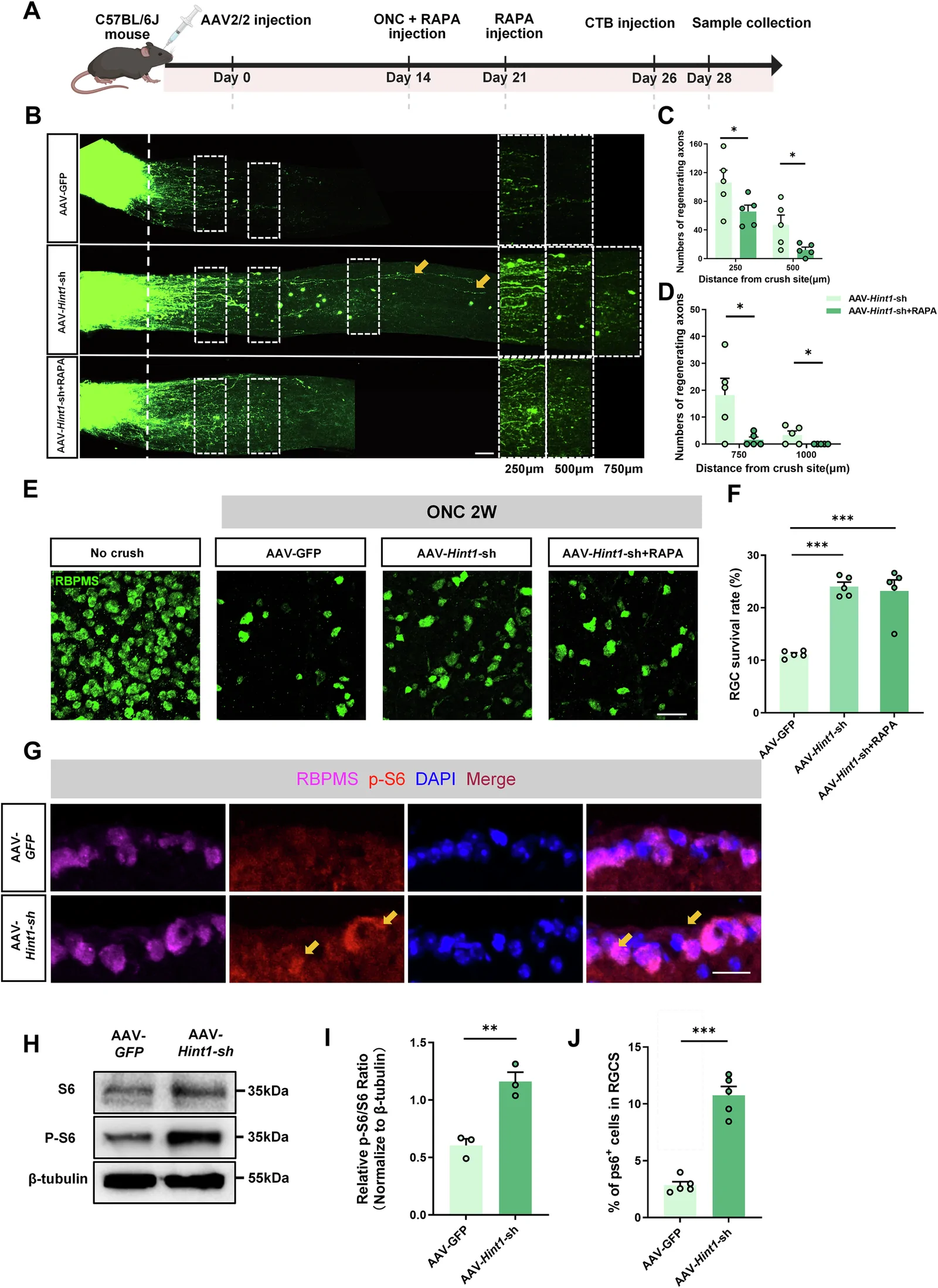
*Hint1* knockdown activates the mTOR pathway to promote neuroprotection and axon regeneration after ONC. **A** Experimental timeline of ONC with intravitreal AAV and RAPA treatment. Mice received intravitreal injection of AAV-GFP or AAV-*Hint1*-shRNA on Day 0, followed by ONC and RAPA injection on Day 14, a second RAPA injection on Day 21, CTB-555 injection on Day 26, and tissue harvest on Day 28. **B** Representative images of CTB-555 labeled axons at 14 days post-ONC. Knockdown of *Hint1* markedly enhanced axon regeneration compared with the control group, whereas co-treatment with RAPA suppressed this effect. The dashed line indicates the crush site; insets show regenerating axons at 250–750 μm distal to the lesion. Scale bar: 100 μm. **C**, **D** Quantification of regenerating axons at increasing distances from the crush site in the AAV-*Hint1*-shRNA and AAV-*Hint1*-shRNA + RAPA groups (*n* = 5 animals, data are means ± SEM. Unpaired two-tailed t test, *p < 0.05). **E** Representative images of RBPMS-immunolabeled (green) RGCs in uninjured, AAV-GFP, AAV-*Hint1*-shRNA, and AAV-*Hint1*-shRNA + RAPA treated retinas at 14 days post-ONC. Scale bar: 50 μm. **F** Quantification of RGC survival at 14 days post-ONC, showing that AAV-*Hint1*-shRNA significantly promoted RGC survival, and co-treatment with RAPA did not reduce this effect (*n* = 5 animals, data are means ± SEM. One-way ANOVA followed by Tukey’s multiple comparisons test, ***p < 0.001). **G** Immunofluorescence images of retinal sections showing RBPMS (magenta), pS6 (red), DAPI (blue), and merged channels in AAV-GFP and AAV-*Hint1*-shRNA treated retinas. Scale bar: 50 μm. **H** Representative western blot showing the expression levels of P-S6, total S6, and β-tubulin in N2a cells treated with AAV-GFP or AAV-*Hint1*-shRNA. **I** Quantification of the relative P-S6/S6 ratio (normalized to β-tubulin) in the samples from (**H**) (*n* = 3 independent experiments, data are means ± SEM. Unpaired two-tailed *t* test, *p < 0.05). **J** Quantification of pS6 positive RGCs in the RGC layer at 14 days post-ONC, demonstrating elevated pS6 expression in AAV-*Hint1*-shRNA treated retinas compared to AAV-GFP controls (*n* = 4 animals, data are means ± SEM. Unpaired two-tailed *t* test, ***p < 0.001).

To confirm that *Hint1* knockdown promotes axon regeneration via the mTOR pathway, we performed in vivo experiments using the mTOR inhibitor rapamycin (RAPA) (Fig. 5A). *Hint1* knockdown alone significantly enhanced axon regeneration, especially in the distal region (250–750 μm) from the crush site (Fig. 5B–D). Co-treatment with RAPA markedly reduced this regenerative effect. *Hint1* knockdown also increased RGC survival, which was not diminished by RAPA (Fig. 5E, F). It is worth noting that RAPA itself also provides some protection to RGC survival [47–49], which may explain why it did not reduce the protective effect of PR-619 on RGC survival. Immunofluorescence analysis further confirmed that mTOR pathway activation was not significantly increased in the AAV-GFP control group, while in the AAV-*Hint1*-shRNA group, the number of p-S6 positive RGCs significantly increased, indicating that mTOR pathway activation was significantly enhanced following *Hint1* knockdown (Fig. 5G, J). Western blot analysis suggested that knockdown of *Hint1* increased p-S6 levels in N2a cells (Fig. 5H, I). Mechanistically, *Hint1* knockdown elevated phosphorylation of mTOR and AKT without affecting total protein levels (Fig. S10B–G). In contrast, the phosphorylation levels of AMPK and ERK, two classic signaling molecules involved in mTOR regulation, showed no significant differences (Fig. S10B–G). Together, our results demonstrate that PR-619 promotes axon regeneration after ONC by reducing HINT1 stability and thereby activating the mTOR pathway.

### Combined therapeutic strategies enhance optic nerve regeneration after ONC

**Fig. 6:**
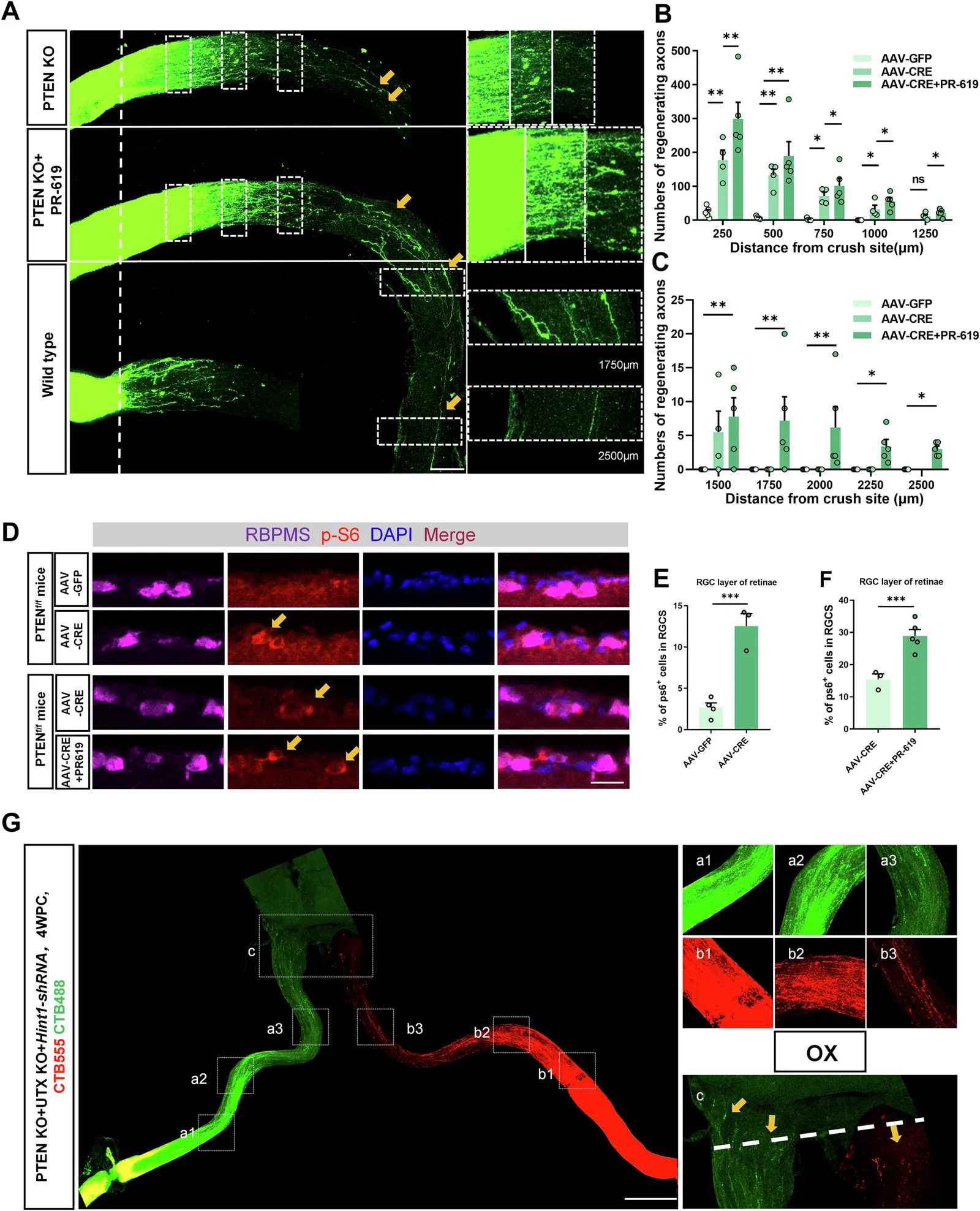
Combined therapeutic strategies enhance axon regeneration after ONC. **A** Representative images of regenerating axons in the optic nerve at 14 days post-ONC. Compared to AAV-GFP and AAV-CRE controls, combined *Pten* knockout and PR-619 treatment significantly enhanced axon regeneration. Dashed line indicates the crush site; insets show magnified images of regenerating axons at various distances distal to the lesion. Scale bar: 250 μm. **B** Quantification of regenerating axons at increasing distances from the crush site. The number of regenerating axons was significantly higher in the AAV-CRE + PR-619 group compared to the AAV-GFP and AAV-CRE groups, especially within 250–1250 μm from the lesion site (*n* = 5 animals per group, data are means ± SEM, one-way ANOVA with Tukey’s multiple comparisons test, *p < 0.05, **p < 0.01, ns no significant). **C** Quantification of regenerating axons at greater distances from the lesion site (1500–2500 μm), showing a marked increase in axon regeneration with combined *Pten* knockout and PR-619 treatment compared to controls (*n* = 5 animals per group, data are means ± SEM, one-way ANOVA with Tukey’s multiple comparisons test, ns not significant, *p < 0.05, **p < 0.01). **D** Representative immunofluorescence images showing RBPMS (magenta) and pS6 (red) in RGCs from *Pten* KO and wild-type mice treated with AAV-CRE or AAV-GFP, with or without PR-619. Scale bar: 50 μm. **E** Quantification of the percentage of pS6 positive RGCs in the RGCs layer, showing significantly increased mTOR pathway activation in PR-619-treated groups, particularly in the *Pten* KO + PR-619 group (*n* = 3–4 animals, data are means ± SEM, unpaired two-tailed t test, ***p < 0.001). **F** Quantification of the percentage of pS6 positive RGCs in the RGCs layer of *Pten* KO mice, showing enhanced activation of the mTOR pathway with combined PR-619 and *Pten* knockout treatment compared to controls (*n* = 5 animals, data are means ± SEM. Unpaired two-tailed *t* test, ***p < 0.001). **G** Representative images of optic nerves at 4 weeks post-crush (4 wpc) in *Utx*^*f/f*^ */ Pten*^*f/f*^ mice treated with AAV-*Hint1*-shRNA and AAV2-Cre. Bilateral anterograde tracing with CTB-555 (red) and CTB-488 (green) was used to label regenerating axons from both optic nerves. Higher-magnification views of the boxed regions (a1–a3, b1–b3) are shown on the right. Scale bar = 500 μm.

To investigate the synergistic effects of *Pten* knockout and PR-619 on axon regeneration, optic nerve crush was performed in *Pten* knockout and wild-type mice, followed by combined AAV-CRE and PR-619 therapy. CTB-555 labeling revealed significantly enhanced axon regeneration in the *Pten* knockout + PR-619 group compared to AAV-GFP and AAV-CRE controls (Fig. 6A). Quantitative analysis showed a marked increase in regenerating axons within the 250–1250 μm region distal to the crush site, particularly prominent in the AAV-CRE + PR-619 group with statistically significant differences versus controls (Fig. 6B). At greater distances (1500–2500 μm), regenerating axons in the combined therapy group remained significantly higher than in controls, indicating that *Pten* knockout combined with PR-619 notably enhanced long-distance axon regeneration (Fig. 6C).

Further analysis assessed mTOR pathway activation in RGCs following *Pten* deletion. *Pten* knockout mice showed a significantly increased proportion of p-S6 positive RGCs, indicating upregulated mTOR activity (Fig. 6D, E). Combined with PR-619, this effect was further enhanced, with a significant increase in p-S6 positive RGCs (Fig. 6D). Quantitative analysis confirmed synergistic mTOR activation by *Pten* knockout and PR-619 (Fig. 6F). Previous studies have shown that the histone demethylase UTX promotes CNS axon regeneration through epigenetic regulation [50]. We therefore hypothesized that simultaneous modulation of UTX and the mTOR pathway might further enhance axonal repair after ONC. To test this hypothesis, we generated *Utx*^*f/f*^/*Pten*^*f/f*^ mice with double knockout of *Utx* and *Pten*, and combined this with AAV-*Hint1*-shRNA treatment. At 4 weeks post-crush, this intervention markedly enhanced optic nerve regeneration (Fig. 6G), with a substantial increase in regenerating axons, some extending to or crossing the optic chiasm. Bilateral tracing with CTB-555 (red) and CTB-488 (green) confirmed robust long-distance axonal growth. Enlarged views (a1–a3 and b1–b3) illustrate regenerating axon distribution in both optic nerves, while magnified panel c highlights axons traversing the chiasmatic boundary.

## Discussion

We first discovered that PR-619 exhibited significant advantages in promoting neurite outgrowth in vitro. High concentrations (>100 μM) caused a decrease in RGC survival, while 10 μM was determined to be the optimal dose in the ONC model. At this concentration, PR-619 effectively promoted axon regeneration while also significantly protecting RGC survival. In long-term treatment experiments, we observed that axon regeneration length gradually increased over time, but its protective effect on RGCs decreased at 4 weeks, suggesting that PR-619 provides strong neuroprotection in the acute phase but has limited long-term effects. Most current gene- and small molecule-based therapies for promoting RGC survival after optic nerve injury serve to delay rather than prevent RGC death. For instance, therapeutic strategies targeting CamKII and M1 [17, 51] have demonstrated that RGC survival rates in treated groups continue to decline during the later stages of injury. Our data similarly indicate that PR-619 delays RGC death following injury. However, the delaying effect is only observable within the first two weeks post-injury. This limitation may arise from the broad-spectrum inhibitory nature of PR-619, which simultaneously affects multiple cell types and proteins, potentially exerting differential effects during the acute and subacute phases of injury [52, 53].

Mechanistically, PR-619 significantly activated the mTOR signaling pathway, with the proportion of p-S6 positive RGCs increasing by over 10%. However, this effect was markedly reduced when combined with the mTOR inhibitor rapamycin (RAPA). Our proteomic data (Fig. 2) revealed a substantial number of differentially downregulated proteins following PR-619 treatment. Although rescue assays with HINT1 overexpression (Fig. 3G, H and S9) strongly confirmed that HINT1 is the major mediator of its effects, other targets may also contribute to the observed regenerative and neuroprotective phenotypes. As a member of the DUB family, USP7 is a potential target of PR-619 [54, 55], its downregulation can modulate the ubiquitination and degradation of pro-apoptotic proteins such as p53 and regulate autophagic flux, which synergizes with PR-619-enhanced parkin-mediated mitophagy to maintain mitochondrial quality control [56]. HDAC4, a key epigenetic regulator of axon regeneration, can relieve the transcriptional repression of axon growth-related genes via its downregulation, potentially promoting optic nerve regeneration [57, 58].

Located in the susceptibility region of the 15q13.3 deletion syndrome, OTUD7A has been established as an independent neurodevelopmental disorder risk gene in recent genetic studies [59–61], emphasizing its key role in nervous system development [62–64]. *Otud7a* knockdown led to a reduction in HINT1 expression, thereby relieving the inhibition of mTOR pathway activity and ultimately promoting axonal regeneration and neuroprotection.

HINT1 is a highly conserved protein involved in various biological processes, including signal transduction, gene expression regulation, and apoptosis [43, 44, 65]. As a classic tumor suppressor protein, HINT1 plays a crucial regulatory role in the development of various malignancies [66, 67], and mutations in its gene can lead to neurodevelopmental disorders. Further mechanistic studies showed that downregulation of *Hint1* relieved its suppression on the mTOR pathway, thereby promoting regeneration and enhancing neuronal regenerative capacity. Notably, previous studies have shown that another family member, HINT3, suppresses AKT/mTOR signaling by promoting *Pten* transcriptional activity, thus inhibiting tumorigenesis [68]. This analogy suggests that HINT1 may similarly function as a negative regulator in CNS injury repair by maintaining mTOR pathway suppression, thereby limiting neuronal regenerative capacity. The hypothesis that HINT1 and HINT3 share analogous inhibitory mechanisms warrants further experimental validation.

Building upon these insights, we further explored the potential of combined drug and gene interventions in promoting CNS regeneration. PTEN, a classic inhibitor of the mTOR pathway, is well-established to significantly enhance neuronal regeneration when deleted [12, 13, 69]. In this study, we induced *Pten* deletion using AAV-CRE and combined it with the DUB inhibitor PR-619, achieving a synergistic effect in promoting axon regeneration. We constructed a more aggressive multi-gene strategy, including the dual knockout of *Pten* and *Utx* and the silencing of *Hint1*, aiming to achieve more pronounced regeneration potential after optic nerve injury. Preliminary results show that this combination strategy induces long-distance axon regeneration extending across the optic chiasm. This discovery provides crucial insights to overcoming the “distance limitation” in CNS regeneration and achieving true functional reconstruction [17, 70, 71]. OTUD7A-specific inhibitors are promising for the precise modulation of neuronal survival and axon regeneration via the targeted regulation of the ubiquitin-proteasome system, yet their clinical translation faces key challenges. These include the need to achieve high selectivity to avoid off-target effects on other DUB family members critical for maintaining normal cellular homeostasis, as well as the difficulty of enabling efficient ocular delivery to ensure the drug reaches retinal ganglion cells and the optic nerve with adequate bioavailability. Both strategies hold unique translational potential, and their further development will require overcoming the respective clinical hurdles involved.

## Supplementary information


Supplemental information manuscript
Original WB data


## Data Availability

The original LC-MS data in this paper have been deposited in the OMIX (OMIX011906), China National Center for Bioinformation / Beijing Institute of Genomics, Chinese Academy of Sciences. Values for data presented in Supplementary Figs. 1–10 are provided in the Supporting Data Values file.
