# Supplementary material for: Targeting OTUD7A-HINT1 deubiquitination activates mTOR signaling for CNS regeneration
Source: Cell Death Dis. 2026 Jun 20;17(1):767. doi: 10.1038/s41419-026-09005-4 (PMC13530147; doi:10.1038/s41419-026-09005-4)
Supplement: Supplementary file 1 [file 41419_2026_9005_MOESM1_ESM.docx]

**Supplemental information**

**Targeting OTUD7A-HINT1 deubiquitination activates mTOR signaling for CNS regeneration**

Zhen-Gang Liu^1,2,*^, Yong-Quan Sun^2,3,4,*^, Lai-Yang Zhou^2,3,4,*^, Ze-Yu Liu^1^, Ling-Wei Zhao^2,3,4^, Feng-Quan Zhou^5,#^, Bo-Yin Zhang^1,#^, Chang-Mei Liu ^2,3,4,6,#^

1.Department of Orthopaedics, China-Japan Union Hospital of Jilin University, Changchun 130033, China

2.Key Laboratory of Organ Regeneration and Reconstruction, Institute of Zoology, Chinese Academy of Sciences, Beijing, 100101, China.

3.University of Chinese Academy of Sciences, Beijing, 100049, China.

4.Beijing Institute for Stem Cell and Regenerative Medicine Beijing, 100101, China.

5.Center for Translational Neural Regeneration Research, Sir Run Run Shaw Hospital, Zhejiang University School of Medicine, Hangzhou, Zhejiang 310016, China.

6.Lead contact

**#**Correspondence author: C-M.L. ([liuchm@ioz.ac.cn](mailto:liuchm@ioz.ac.cn)), B-Y.Z ([drboyin@jlu.edu.cn](mailto:drboyin@jlu.edu.cn)) and F-Q.Z. (fzhou4@zju.edu.cn)

*These authors contributed equally to this paper.

**Keywords:** Ubiquitination, PR-619, OTUD7A, HINT1, Central nervous system, Axon regeneration, Optic nerve crush, Spinal cord injury

**Table S1. Primer’s sequences**

| Gene | Forward | Reverse |
| --- | --- | --- |
| shRNA sequences | | |
| *Otud7a* | GGCCTACGATCAAGCTCACTTTTCAAGAGAAAGTGAGCTTGATCGTAGGCTTTTTATTT | |
| *Hint1* | GGCAAGATCATCCGCAAAGAAATCAAGAGTTTCTTTGCGGATGATCTTGCTTTTTATTT | |
| *Scramble* | TTATTGCTTAAGAATACGCGTAGTTCAAGAGACTACGCGTATTCTTAAGCAATAATTTTT | |
| RT-PCR primers | | |
| *Otud7a* | GTCTCTAGCCTGCTTCCAAAC | GTGGACCGAACAAAGTCTGAC |
| *Hint1* | GCGACACGATCTTCGGCAA | GGTGCTTGAGGGGAAATGTCA |
| *Gapdh* | TGTGATGGGTGTGAACCACGAGAA | CTGTGGTCATGAGCCCTTCCACAA |
| Amplified by PCR | | |
| *Otud7a* | CCGTTTAAACATGGTCTCTAGCCTGCTTCCAAAC | CCTCGAAGGTAGAACCTCGCC |
| *Otud7a ^Flag^* | ATGGTCTCTAGCCTGCTTCC | CGCGCGCGGCGCGACCCACGCGTACGCGGCCGCTCGAGCAGAAACTCATCTCAGAAGAGGATCTGGCAGCAAATGATATCCTGGATTACAAGGATGACGACGATAAG |
| *Hint1* | GCGTTTAAACATGGCTGACG | AGATGAACTGGCCTCCTGGT |
| *Hint1 ^His^* | GCGTTTAAACATGGCTGACG | GCCTCCTGGTCATCATCACCATCACCAT |
| *Hint ^K7R^* | GCGTTTAAACATGGCTGACGAGATTGCCAGGGC | CCCAAGCTTTTAATGGTGATGGTGAT |
| *Hint ^K21R^* | GACACGATCTTCGGCAGGATC | GATCCTGCCGAAGATCGTGTC |
| *Hint ^K25R^* | CGGGCAGACAGTTGCGTAAAGAGTCCCAGAAAGACCGCAA | TTTACGCAACTGTCTGCCCG |
| *Hint ^K30R^* | ATCCCCGCCAGGATCATCTT | AAGATGATCCTGGCGGGGAT |
| *Hint ^K57R^* | CTGGTGATACCCAGGAAGCA | TGCTTCCTGGGTATCACCAG |
| *Hint ^K58R^* | CTGGTGATACCCAAGAGGCA | TGCCTCTTGGGTATCACCAG |
| *Hint ^K82R^* | GTTGGCAGGAAATGTGCTGC | GCAGCACATTTCCTGCCAAC |
| *Hint ^K83R^* | GTTGGCAAGAGATGTGCTGC | GCAGCACATCTCTTGCCAAC |
| *Hint ^K91R^* | CAGATCTGGGCCTGAGGCGC | GCGCCTCAGGCCCAGATCTG |

*Italics indicate the mouse gene name

**Graphical Abstract**

**KEY RESOURCES TABLE**

| **REAGENT or RESOURCE** | **SOURCE** | **IDENTIFIER** |
| --- | --- | --- |
| **Antibodies** | | |
| Mouse anti-Tubulin b3(Tuj1) | Biolegend | Cat# ab801202, RRID: AB_10063408 |
| Rabbit anti-RBPMS | Abcam | Cat# ab194213, RRID: AB_2920590 |
| Guinea pig anti-RBPMS | PhosphoSolutions | Cat# 1832-RBPMS, RRID: AB_2492226 |
| Rabbit anti-HINT1 | Abmart | Cat# T510670, RRID: AB_124912 |
| Alexa Fluor 488 AffiniPure Donkey Anti-Mouse IgG(H+L) | Thermo Fisher Scientific | Cat# A-21202, RRID: AB_141607 |
| Alexa Fluor 647 AffiniPure Donkey Anti-Mouse IgG(H+L) | Thermo Fisher Scientific | Cat# A-31571, RRID: AB_162542 |
| Alexa Fluor 488 AffiniPure Donkey Anti-Rabbit IgG(H+L) | Thermo Fisher Scientific | Cat# A-21206, RRID: AB_2535792 |
| Alexa Fluor 647 AffiniPure Donkey Anti-Rabbit IgG(H+L) | Thermo Fisher Scientific | Cat# A-31573, RRID: AB_2536183 |
| Alexa Fluor 488 AffiniPure Goat Anti-Guineapig IgG(H+L) | Thermo Fisher Scientific | Cat# A-11073, RRID: AB_2534117 |
| anti-Flag-Tag Mouse Monoclonal Antibody | Easybio | Cat# BE2072-10 |
| anti-His-Tag Mouse Monoclonal Antibody | Easybio | Cat# BE2006 |
| **Chemicals,peptides,and recombinant proteins** | | |
| CTB,Alexa Fluor 555 Conjugate | Thermo Fisher Scientific | Cat# C34775 |
| N-Methyl-D-aspartic Acid (NMDA) | Millipore Sigma | Cat# 454575 |
| Dynabeads M-450 Epoxy | Thermo Fisher Scientific | Cat# 14011 |
| MEM | Gibco | Cat# 11140050 |
| DMEM/F12(1:1) | Gibco | Cat# 11320033 |
| Neurobasal | Gibco | Cat# 21103049 |
| Opti-MEM reduced serum medium | Thermo Fisher Scientific | Cat# 31985-070 |
| GlutaMAX-I | Gibco | Cat# 35050061 |
| HBSS | Thermo Fisher Scientific | Cat# 14175-095 |
| Penicillin-Streptomycin | Hyclone | Cat# SV30010 |
| B-27（50X） | Gibco | Cat# 12587010 |
| T4 DNA Ligase | NEB | Cat# M0202V |
| XbaI | NEB | Cat# R0145S |
| BamHI | NEB | Cat# R0136V |
| BsaI-HF | NEB | Cat# R3535S |
| Benzyl benzoate ReagentPlus | Sigma-Aldrich | Cat# B6630 |
| Benzyl alcohol anhydrous | Sigma-Aldrich | Cat# 305197 |
| T48402-25G,2,2,2-Tribromoethanol | Sigma-Aldrich | Cat# T48402 |
| Lipofectamine 2000 | Life Technologies | Cat# 11668019 |
| **Critical commercial assays** | | |
| FastPure Gel DNA Extraction Mini Kit | Vazyme | DC301-01 |
| 2×Phanta® Max MasterMix | Vazyme | P515-01 |
| Papain Dissociation System | Worthington | LK003150 |
| **Experimental models: Cell lines** | | |
| AAV-293 | Procell | CL-0019 |
| Neuro-2a | This study | N/A |
| **Experimental models: Organisms/strains** | | |
| Mouse:C57BL/6J | SiPeiFu（Beijing） | B204-02 |
| Mouse: *Kdm6a^flox/flox^* | Jackson Labs | B6.129S4-Ptentm1Hwu/J |
| Mouse: *Pten^flox/flox^* | Jackson Labs | Kdm6atm1c(EUCOMM)Jae |
| **Oligonucleotides** | | |
| For all primers,see Table S1 | This study | N/A |
| **Recombinant DNA** | | |
| AAV2-CAG-GFP | This study | N/A |
| AAV2-CAG-PLAP | This study | N/A |
| AAV2-CAG-*Hint1*-shRNA | This study | N/A |
| AAV2-CAG-*Otud7a*-shRNA | This study | N/A |
| AAV2-CAG-*Hint1* | This study | N/A |
| **Software and algorithms** | | |
| Prism | GraphPad | RRID:SCR_002798 |
| ImageJ | NIH | RRID:SCR_003070 |
| LASX | Leica | N/A |
| ZEN 2009 Light Edition | Zeiss | N/A |
| **Other** | | |
| Zeiss LSM 880 Fast Ariyscan | Zeiss | N/A |
| BD FACS Fusion | BD Biosciences | N/A |
| LEICA DMi8 | Leica | N/A |

**Fig.S1 PR-619 promotes neurite growth in cortical neurons and retinal explants.**

(A) Experimental timeline. Primary cortical neurons and retinal explants were isolated from postnatal day 0 (P0) mice. Cortical neurons were treated with PR-619 on day 5 in vitro and collected on day 7. Retinal explants were treated with PR-619 on day 3 or 5 and harvested on day 5 or 7 for fixation and immunostaining.

(B) Representative images of primary cortical neurons from control group and different concentrations of PR-619 groups, showing neurite morphology. Neurons were labeled with MAP2 (red) and nuclei were stained with DAPI (blue). Scale bar: 100 μm.

(C) Representative images of neurite growth in retinal explants from control and PR-619-treated groups on day 5 and day 7. Dashed boxes indicate magnified areas showing regenerating neurites. Scale bar: 100 μm.

(D) Quantification of total neurite length in cortical neurons. PR-619 (0.5 μM) treatment significantly increased the maximal total neurite length compared to DMSO-treated groups (n = 3 independent experiments, n numbers on graph show the number of analysed cells, data are means ± SEM, One-way ANOVA followed by Tukey’s multiple comparisons test, ns = no significant, **p* < 0.05, ****p* < 0.001).

(E) Quantification of neurite number in retinal explants on day 5. Compared with DMSO-treated groups, PR-619 treatment significantly increased neurite growth in the 50-100 μm and 100-200 μm ranges (n = 5-6 animals, data are means ± SEM. Unpaired two-tailed t test, ns = no significant, **p* < 0.05).

(F) Quantification of neurite number in retinal explants on day 7. Compared with DMSO-treated groups, PR-619 treatment significantly enhanced neurite growth (n = 5-6 animals, data are mean ± SEM, unpaired two-tailed t-test, **p* < 0.05).

**Fig.S2** **MG132 does not promote neurite growth, axon regeneration, or RGC survival after ONC.**

(A) Experimental timeline. Mice received ONC and MG132 injection on Day 0, followed by MG132 injection on Day 7, CTB injection on Day 12, and tissue harvest on Day 14.

(B) Representative images of MAP2 (red) and DAPI (blue) staining in primary cortical neurons treated with DMSO or MG132. No significant differences in neurite growth were observed between the DMSO and MG132 treatment groups. Scale bar: 100 μm.

(C) Quantification of total neurite length in cortical neurons treated with DMSO or MG132, showing no significant difference between the two groups (n = 4 independent experiments, data are means ± SEM, unpaired two-tailed t test, ns = no significant).

(D) Representative images of CTB-labeled regenerating axons in the optic nerve at 2 weeks post-ONC, showing no significant difference between MG132 and DMSO treatment groups. Dashed line indicates the crush site. Scale bar: 100 μm.

(E) Quantification of regenerating axons at increasing distances (250-1000 μm) distal to the crush site, showing no significant increase in axon regeneration with MG132 treatment compared to DMSO controls (n = 5-6 animals, data are means ± SEM, one-way ANOVA followed by Tukey’s post hoc test, ns = not significant).

(F) Representative images of RBPMS-labeled RGCs at 2 weeks post-ONC in uninjured and ONC groups treated with DMSO or MG132, showing no significant difference in RGC survival between the groups. Scale bar: 100 μm.

(G) Quantification of RGC survival at 2 weeks post-ONC, showing no significant difference in survival between MG132 and DMSO groups (n = 6 animals, data are means ± SEM, unpaired two-tailed t test, ns = no significant).

**Fig.S3** **Determination of the safe concentration range of PR-619 in RGCs and its inhibitory effect on deubiquitinases.**

(A) Experimental timeline. Mice received PR-619 injection on Day 0, followed by sample collection on Day 7.

(B) Representative images of wholemounts stained for RBPMS (green) to label RGCs in the retinas 1 week after PR-619 injection at various concentrations (5-200 µM). Scale bar: 100 µm.

(C) Quantification of RGC survival 1 week post-PR-619 injection. PR-619 treatment at concentrations of 5-50 µM did not affect RGC survival compared to the DMSO control, while concentrations of 100-200 µM significantly reduced RGC survival (n = 3-5 animals, data are means ± SEM, one-way ANOVA followed by Tukey’s multiple comparisons test, ns = no significant, **p* < 0.05, ****p* < 0.001).

(D) Representative immunofluorescence images of retinal sections co-stained for RBPMS (red), ubiquitin (magenta), and DAPI (blue) in DMSO and PR-619 treated retinas. Scale bar: 50 µm.

(E) Quantitative analysis of UB fluorescence intensity in RBPMS positive RGCs, PR-619 treatment significantly increased UB fluorescence intensity in RGCs compared to DMSO control (n = 3 animals, data are means ± SEM. Unpaired two-tailed t test, ****p* < 0.001).

(F) Representative immunofluorescence images of retinal sections showing the distribution of UB (magenta) in the inner nuclear layer (INL) of DMSO and PR-619 treated retinas, RBPMS (red) labels RGCs in the ganglion cell layer (GCL), and DAPI (blue) stains nuclei. Scale bar: 50 µm.

(G) Quantification of UB fluorescence intensity in the INL, no significant difference was observed between DMSO and PR-619 groups (n = 3 animals, data are means ± SEM, unpaired two-tailed t test, ns = no significant).

**Fig.S4** **PR-619 treatment does not augment microglial activation after ONC.**

Representative images of retinal sections showing the inflammatory response of the retina to PR-619 treatment after ONC. Retinal sections were labeled with TUJ1 (red) to mark neurons, CD68 (green) and IBA1 (white) to label microglia/macrophages, and DAPI (blue) for nuclei staining. Scale bar: 50 µm.

**Fig.S5 PR-619 provides short-term protection of RGC survival but fails to protect long-term after ONC.**

(A) Representative images of RGCs labeled with RBPMS (green) at 2 weeks (ONC 2W) and 4 weeks (ONC 4W) post-ONC, with PR-619 or DMSO treatment. Scale bar: 100 µm.

(B) Quantification of RGC survival at 2 and 4 weeks post-ONC. PR-619 significantly increased RGC survival at 2 weeks compared to DMSO, but did not have an effect at 4 weeks post-ONC (n = 5 animals, data are means ± SEM, one-way ANOVA followed by Tukey’s post hoc test, ns = no significant, ****p* < 0.001).

(C) Experimental timeline for NMDA-induced retinal injury with PR-619 treatment. Mice received NMDA and PR-619 injection on Day 0, PR-619 injection on Day 7, and tissue harvest on Day 14.

(D) Representative RBPMS-labeled RGCs (green) in uninjured, DMSO-treated, and PR-619-treated retinas at 14 days post-NMDA injury. Scale bar: 50 μm

(E) Quantification of RGC survival in NMDA-induced retinal injury, showing significantly higher survival in PR-619-treated retinas compared to DMSO (n = 3 animals, data are means ± SEM. Unpaired two-tailed t test, ****p* < 0.001).

**Fig.S6 PR-619 promotes neural network reconstruction after spinal cord injury.**

(A) Experimental timeline: C57BL/6J mice were subjected to SCI, followed by the first injection of PR-619 at Day 0. PR-619 was administered once daily thereafter. Behavioral assessments, including BMS scoring and inclined plane test, were performed from Day 7, and tissue samples were collected at Day 35.

(B) Histological staining: Representative sagittal sections of Sham, SCI, and SCI+PR-619 groups stained with HE, Nissl, and LFB. Upper panels show low-magnification overviews,Scale bar: 1000 μm. and boxed regions indicate enlarged views in a1–a3, b1–b3, and c1–c3. Scale bar: 100 μm.SCI mice exhibited prominent cavitation, disrupted tissue architecture, neuronal loss, and severe demyelination. PR-619 treatment reduced lesion size, preserved structural continuity, increased surviving neurons with intact Nissl bodies, and improved myelin retention.

(C) Masson’s trichrome staining: Skeletal muscle from the hindlimb exhibited marked fiber atrophy and fibrosis after SCI, which were attenuated by PR-619 treatment. Scale bar: 50 μm.

(D) BMS locomotor scores: PR-619 markedly improved motor recovery versus the SCI group, with significantly higher BMS scores; improvements were evident from Day 7 and sustained through Day 35.

(E) Inclined plane test: PR-619 increased the maximum supporting angle compared with the SCI group, with benefits appearing by Day 7 and persisting through Day 35 (Data are presented as mean ± SEM; n = 6 animals. One-way ANOVA followed by Tukey’s multiple comparisons test; ns = not significant, **p < 0.01, ***p < 0.001, ****p < 0.0001).

**Fig.S7 PR-619 activates the mTOR pathway in RGCs but does not alter GSK3β or STAT3 signaling.**

(A) Representative immunofluorescence images of retinal sections showing RBPMS (magenta), pS6 (red), and DAPI (blue) between the DMSO and PR-619 treated mice. Scale bar：50 μm

(B) Representative images of retinal sections showing that PR-619 treatment did not alter GSK3β phosphorylation (p-GSK3β) in RGCs 3 days after injection. Retinal sections were stained with RBPMS (red), p-GSK3β (green), and DAPI (blue). Scale bar: 50 μm.

(C) Representative images of retinal sections showing that PR-619 treatment did not alter STAT3 phosphorylation (p-STAT3) in RGCs 3 days after injection. Retinal sections were stained with RBPMS (red), p-STAT3 (green), and DAPI (blue). Scale bar: 50 μm.

(D) Quantification of pS6-positive RGCs, showing significantly increased mTOR pathway activity between the DMSO and PR-619 treated groups (n = 5 animals, data are means ± SEM. Unpaired two-tailed t test, ****p* < 0.001).

(E) Quantification of the relative fluorescence intensity of p-GSK3β in RGCs from (A). No significant differences were observed between the DMSO and PR-619 treated groups (n = 3 animals, data are means ± SEM, unpaired two-tailed t test, ns = no significant).

(F) Quantification of the relative fluorescence intensity of p-STAT3 in RGCs from (B). No significant differences were observed between the DMSO and PR-619 treated groups (n = 3 animals, data are means ± SEM, unpaired two-tailed t test, ns = no significant).

**Fig.S8 AAV2 transduction efficiency and verification in RGCs.**

(A) Representative images of retinal sections showing AAV2 transduction efficiency in RGCs, labeled with the specific RGC marker RBPMS (red) and AAV2 (green). Scale bar: 50 µm.

(B) Representative images of wholemount retinas showing AAV2 transduction efficiency, with staining for RBPMS (red) and AAV2 (green), verifying the infection of RGCs. Scale bar: 50 µm.

(C) Quantification of AAV2 transduction efficiency in RGCs. The transduction efficiency is calculated based on the number of GFP-positive RGCs (GFP+), RBPMS+RGCs, and the total number of RBPMS+ cells (n = 5 animals, data are means ± SEM).

(D) Quantification of *Hint1* mRNA expression levels after knockdown (shRNA) in RGCs, showing significant reduction compared to controls (n = 3 independent experiments, data are means ± SEM, unpaired two-tailed t test, ****p* < 0.001).

(E) Quantification of *Hint1* mRNA expression levels following overexpression in RGCs, showing a significant increase compared to controls (n = 3 animals, data are means ± SEM. Unpaired two-tailed t test, ****p* < 0.001).

(F) Quantification of *Otud7a* mRNA expression levels after knockdown (shRNA), showing a significant reduction compared to controls (n = 3 independent experiments, data are means ± SEM, unpaired two-tailed t test, **p* < 0.05).

(G) Representative immunofluorescence images of retinal sections stained for RBPMS (red), HINT1 (magenta), and DAPI (blue) in retinas transduced with AAV-GFP, AAV-*Hint1*-sh, or AAV-*Hint1*. Scale bar: 50 µm.

(H) Quantitative analysis of HINT1 fluorescence intensity in RBPMS positive RGCs, HINT1 expression was significantly decreased in the AAV-*Hint1*-sh group and increased in the AAV-*Hint1* group compared to the AAV-GFP control (n = 3 animals, n numbers on graph show the number of analyzed cells, data are means ± SEM, unpaired two-tailed t test, ****p* < 0.001).

(I) Representative immunofluorescence images of retinal sections stained for RBPMS (red), OTUD7A (magenta), and DAPI (blue) in retinas transduced with AAV-GFP or AAV-*Otud7a*-sh. Scale bar: 50 µm.

(J) Quantitative analysis of OTUD7A fluorescence intensity in RBPMS-positive RGCs, OTUD7A expression was significantly reduced in the AAV-*Otud7a*-sh group compared to the AAV-GFP control (n = 3 animals, n numbers on graph show the number of analyzed cells, data are means ± SEM, unpaired two-tailed t test, ****p* < 0.001).

**Fig.S9 *Hint1* overexpression attenuates the effect of PR-619 on neurite growth in cultured neurons.**

(A) Representative images of primary cortical neurons transduced with AAV-GFP, AAV-*Hint1*-shRNA, AAV-GFP + PR-619, and AAV-*Hint1* + PR-619, labeled with MAP2 (red), DAPI (blue for nuclei staining), and GFP (green for transduction). Scale bar: 50 µm.

(B) Quantification of total neurite length in primary cortical neurons after treatment with different AAV2 vectors and PR-619. Neurons overexpressing *Hint1* showed reduced neurite growth compared to controls and PR-619 treatment, indicating that *Hint1* overexpression attenuated the regenerative effect of PR-619 (n = 3 independent experiments, n numbers on graph show the number of analyzed cells, data are means ± SEM, One-way ANOVA followed by Tukey’s multiple comparisons test, ns = no significant, **p* < 0.05, ***p* < 0.01, ****p* < 0.001).

**Fig.S10 Screening of HINT1 lysine mutants for OTUD7A-regulated ubiquitination sites and validation of HINT1-mediated mTOR activation via AKT phosphorylation.**

(A) Immunoblot analysis of OTUD7A-Flag effects on HINT1-His mutant ubiquitination. 293T cells were transfected with OTUD7A-Flag and WT Ub-HA plasmids, along with either WT HINT1-His or various HINT1-His mutants (K21R，K25R，K30R，K57R，K58R，K82R，K83R，K91R)Cells were treated with 10 μM MG132 for 6 hours before collection and ubiquitination assay.

(B) Representative western blot images showing the protein levels of phosphorylated and total mTOR, AKT, AMPK, ERK, S6, and the loading control GAPDH in N2a cells transduced with AAV-GFP or AAV-*Hint1*-sh.

(C) Quantitative analysis of relative P-S6/S6 levels (normalized to GAPDH) in N2a cells following AAV-*Hint1*-sh or AAV-GFP treatment. Data are presented as individual data points with mean ± SEM (n = 3 independent experiments, Unpaired two-tailed t test, ***p* < 0.01).

(D) Quantitative analysis of relative p-mTOR/mTOR levels (normalized to GAPDH) in the indicated groups. Data are presented as individual data points with mean ± SEM (n = 3 independent experiments, Unpaired two-tailed t test, ***p* < 0.01).

(E) Quantitative analysis of relative p-AKT/AKT levels (normalized to GAPDH) in the indicated groups. Data are presented as individual data points with mean ± SEM (n = 3 independent experiments, Unpaired two-tailed t test, ***p* < 0.01).

(F) Quantitative analysis of relative p-ERK/ERK levels (normalized to GAPDH) in the indicated groups. Data are presented as individual data points with mean ± SEM (n = 3 independent experiments, Unpaired two-tailed t test, ns = no significant).

(G) Quantitative analysis of relative p-AMPK/AMPK levels (normalized to GAPDH) in the indicated groups. Data are presented as individual data points with mean ± SEM (n = 3 independent experiments, Unpaired two-tailed t test, ns = no significant).
