# Supplementary material for: Targeting OTUD7A-HINT1 deubiquitination activates mTOR signaling for CNS regeneration
Source: Cell Death Dis. 2026 Jun 20;17(1):767. doi: 10.1038/s41419-026-09005-4 (PMC13530147; doi:10.1038/s41419-026-09005-4)
Supplement: Supplementary file 2 [file 41419_2026_9005_MOESM2_ESM.docx]

**Original WB data**
